# Targeting Epithelial-to-Mesenchymal Transition in Radioresistance: Crosslinked Mechanisms and Strategies

**DOI:** 10.3389/fonc.2022.775238

**Published:** 2022-02-16

**Authors:** Lili Qiao, Yanfei Chen, Ning Liang, Jian Xie, Guodong Deng, Fangjie Chen, Xiaojuan Wang, Fengjun Liu, Yupeng Li, Jiandong Zhang

**Affiliations:** ^1^ Department of Oncology, The First Affiliated Hospital of Shandong First Medical University and Shandong Province Qianfoshan Hospital, Shandong Lung Cancer Institute, Jinan, China; ^2^ Department of Oncology, Shandong First Medical University, Jinan, China; ^3^ Department of General Surgery, The First Affiliated Hospital of Shandong First Medical University, Jinan, China

**Keywords:** EMT, radioresistance, mechanisms, strategies, cancer stem cell, tumor microenvironment

## Abstract

Radiotherapy exerts a crucial role in curing cancer, however, its treatment efficiency is mostly limited due to the presence of radioresistance. Epithelial-to-mesenchymal transition (EMT) is a biological process that endows the cancer cells with invasive and metastatic properties, as well as radioresistance. Many potential mechanisms of EMT-related radioresistance being reported have broaden our cognition, and hint us the importance of an overall understanding of the relationship between EMT and radioresistance. This review focuses on the recent progresses involved in EMT-related mechanisms in regulating radioresistance, irradiation-mediated EMT program, and the intervention strategies to increase tumor radiosensitivity, in order to improve radiotherapy efficiency and clinical outcomes of cancer patients.

## 1 Introduction

Radiotherapy has always been an effective therapy for cancers. More than half of cancer patients are treated with radiotherapy alone or in combination with other treatments. It primarily exerts its role by direct physical damage to DNA or indirect damage from reactive oxygen species. Despite the appreciable advances in radiotherapy in recent years, most cancer patients remain poor prognosis. Tumor radioresistance has been considered as a powerful obstacle to impinge on radiotherapy efficacy, and EMT acts as one of the most important factors involving radioresistance (1, 2). Epithelial-to-mesenchymal transition (EMT) is a biological process that refers to the transition of epithelial cells to ones with mesenchymal phenotype. This process is generally accompanied by the alterations in cell morphology, cell-cell adhesion and cellular signaling factors. It is characterized by the loss of epithelial adhesion markers (E-cadherin) and the gain of mesenchymal markers such as N-cadherin, vimentin, fibronectin and alpha smooth muscle actin (a-SMA) (3).

In this review, we conduct a comprehensive review focusing on the recent advances in EMT-related mechanisms in regulating radioresistance and the influence of irradiation on EMT, and excavate the potential strategies to overcome radioresistance.

## 2 EMT-Mediated Mechanisms in Radioresistance

EMT is known to play a central role in inducing resistance of tumor cells to irradiation, and it is regulated by a variety of molecular mechanisms containing TGF-β, Wnt/β-catenin, PI3K/AKT, Notch, NF-κB, IL-6/STAT3, non-coding RNAs and so on. Also, cancer stem cells and tumor microenvironment participate in the acquisition of EMT program and tumor radioresistance. These mechanisms function mainly depending on the activation of EMT-related transcription factors (TFs) and markers, including Snail/Slug, ZEB1/2, Twist1/2, E-cadherin, N-cadherin, Vimentin (4–7). EMT-related mechanisms in regulating radioresistance are depicted in **Table 1**.

**Table 1:** EMT-mediated mechanisms in radioresistance.

Mechanism	Targeted molecule	Function	Radiotherapy effect	Reference
Signaling pathways	TGF-β	EMT-mediated CSC program; regulate expression of EMT markers	Radioresistance	(8–10)
	Wnt/β-catenin	Modulate EMT-related genes expression; increase ALDH activity	Radioresistance	(11–14)
	PI3K/AKT	Regulate expression of Snail, Twist and EMT markers	Radioresistance	(15–18)
	Notch	Promote the expression of ZEB1, Slug, Snail, NF-κB and Vimentin	Radioresistance	(19–21)
	NF-κB	Regulate Twist, Snail and SIP1	Radioresistance	(22–25)
	IL-6/STAT3	Regulate the expression of Zeb1 and mesenchymal markers	Radioresistance	(26, 27)
	S1PR1	Activate STAT3 and promote CSC program	Radioresistance	(28–30)
Non-coding RNAs	miR-124	Targeting an EMT inducer PRRX1	Radiosensitivity	(31)
	miR-145	Regulate ZEB2 expression	Radiosensitivity	(32)
	miR-205	Regulate PTEN/PI3K/AKT signaling	Radioresistance	(33)
	miR-624-3p	Modulate PTEN/PI3K/AKT signaling	Radiosensitivity	(34)
	miR−301a	Wnt1-dependent EMT	Radiosensitivity	(35)
	LncRNA-TUG1	Target the miR-145/ZEB2 axis	Radioresistance	(32)
	LncRNA-NEAT1	Target the miR-204/ZEB1axis	Radioresistance	(36)
	LncRNA-UCA1	Regulate the expression of MMP-9, ZEB1 and Vimentin	Radioresistance	(37)
Cancer stem cells	EMT-TF	EMT-TF-inducted EMT program provokes the acquisition of tumor-initiating CSCs by regulate stem-cell markers	Radioresistance	(38)
	CD29	Induce EMT phenotype, and facilitate the radioresistance *via* modulating	Radioresistance	(39)
	CD44	ILK-AKT-mTORC1 signaling	Radioresistance	(40, 41)
	TAMs	Drive EMT process *via* PI3K/AKT, Wnt/β-catenin or ERK pathways	Radioresistance	(42–50)
Tumor microenvironment	T cells	Regulate PI3K/AKT, HIF-1α, EGFR/ERK1/2, TGF-β, JAK/STAT signaling pathways	Radioresistance	(51, 52)
	Neutrophils	Facilitate the expression of FN1, VIM, TGM2 and ZEB1; increase the secretion of IL-6	Radioresistance	(53)
	NK cells	Exacerbate hypoxia microenvironment and stabilize Snail	Radioresistance	(54)
	Mast cells	Increase stemness and expression of EMT markers and morphologic switching	Radioresistance	(55)
	Exosomes derived from T cells	Release IL-8 to induce EMT Increase the β-catenin expression and activate the NF-κB/snail pathway	Radioresistance	(56)
	CAFs	Induce paracrine TGF-β; reduce a lot of cytokines and growth factors, such as IL-6, EGF, VEGF and HGF	Radioresistance	(57–60)
	Hypoxia	Activate TGF-β, NF-κB and Notch pathways; promote the expression of Zeb1, Snail and Twist; regulate the expression of EMT markers	Radioresistance	(61–63)

### 2.1 Signaling Pathways in Radioresistance

#### 2.1.1 TGF-β Pathway

The TGF-β family works as an important inducer in EMT program. For instance, the TGF-β, Activin and Nodal, can induce EMT in epithelial cells under different circumstances (64). It was once thought that TGF-β mainly exerted suppressive roles in tumorigenesis due to its ability to inhibit epithelial cell growth and induce cell apoptosis. However, subsequent studies discovered that high levels of TGF-β promoted tumor infiltration and metastasis by inducing EMT. TGF-β acts on target cells through binding to specific receptors, resulting in downstream phosphorylation of Smad2 and Smad3. Phosphorylated Smads form a transcriptional complex with the cofactor Smad4, which enters into the nucleus and binds to specific gene promoters to regulate gene transcription (65). It has been reported that overexpression of Smad2/3 promotes TGF-β-induced EMT, whereas its dominant negative form can abolish the EMT phenotype (66). Besides, the non-Smad-dependent TGF-β pathway is also shown to be associated with EMT process, in which p38 is one of commonly studied molecules. In mouse mammary epithelial NMuMG cells, TGF-β could phosphorylate and activate p38 protein, which is required for EMT process. Synergistic stimulation of TGF-β and TNF-α to induce the morphological alteration from dense epithelial cells to dispersed mesenchymal cells in human colon cancer models is also dependent on enhanced p38 activity (67). In addition, classical protein kinases such as PKA and PKD also play a key role in TGF-β-induced EMT (68, 69). Inhibition of TGF-β pathway strongly blocked the EMT and cancer stem cell (CSC) program, conferring an increase of radiosensitivity (8). In breast cancers, Konge J et al. established the cell model with EMT phenotype induced by TGF-β, they found that these cells acquired the CSC properties, and exhibited enhanced radioresistance (9). Besides, in nasopharyngeal carcinoma (NPC), TGF-β could promote tumor invasion and EMT process by decrease of E-cadherin and increase of Vimentin, thus enhancing the resistance of cancer cells to irradiation (10).

#### 2.1.2 Wnt/β-Catenin Pathway

Activation of Wnt/β-catenin signaling is important in regulating stemness, metastasis, and proliferation of malignant tumors, and recent studies have also shown that it is implicated in tumor radioresistance by inducing EMT (70, 71). Bastos LG et al. (11) reported that Wnt/β-catenin signaling could promote the radioresistance and metastasis of NPC cells by increasing nuclear translocation of β-catenin and transcriptional upregulation of EMT-related genes expression. Another study reported that Wnt/β-catenin pathway triggered the acquisition of EMT phenotype by modulating the activity of aldehyde dehydrogenase (ALDH), thus improving the resistance of prostate cancer progenitor cells to irradiation (12). Inhibition of the Wnt/β-catenin pathway using a β-Catenin/TCF inhibitor FH535, could reverse EMT phenotype and enhance the radiosensitivity of esophageal cancer cells (13). Besides, knockdown of FOXO3a contributed to the transition of EMT phenotype by activating Wnt/β-catenin pathway, thereby resulting in radioresistance (14).

#### 2.1.3 PI3K/AKT Pathway

PI3K/AKT pathway is frequently activated in various human cancers, and has been considered a promising therapeutic target. Increasing studies demonstrated that PI3K/AKT pathway played a crucial role in cell proliferation, survival, metastasis and EMT (72, 73). PI3K/Akt pathway participates in the regulation of Snail, one of the most important EMT-TFs by multiple mechanisms. First, PI3K/Akt activation can promote the phosphorylation of GSK-3β, which accelerates the ubiquitination and degradation of GSK-3β, thus decreasing its degradation to Snail (15). Second, Snail can be directly regulated by PI3K/Akt pathway (16). Twist, another EMT-associated TF, is also positively regulated by PI3K/Akt signaling (16). Several studies have reported that activation of PI3K/Akt pathway is instrumental in enhancement of radioresistance of cancer cells by modulating the expression of EMT markers (17, 18). Besides, in non-small cell lung cancer (NSCLC) cells, PI3K/AKT pathway activated by miR-410/PTEN, functions as an important promotor in occurrence of EMT and radioresistance (74).

#### 2.1.4 Notch Pathway

Notch signaling exerts a crucial role in carcinogenesis by regulating tumor microenvironment or EMT (75, 76). Activation of Notch signaling is triggered by ligand-binding to generate intracellular domain of Notch1 (ICN1), which forms a transcriptional complex with the transcriptional factor CSL/RBPJ and other co−activators such as mastermind-like 1 (MAML1) and EP300, thus promoting the transcription of target genes such as the family of HES/HEY (77). Notch pathway activation contributed to the acquisition of EMT. Conversely, down-regulation of Notch signaling by siRNA could partially reverse the EMT phenotype by decreasing the expression ZEB1, Slug, Snail, NF-κB and Vimentin (19). Furthermore, suppression of Notch signaling by γ-secretase inhibitors or siRNAs could boost the sensitivity of tumor cells to irradiation (20, 21).

#### 2.1.5 NF-κB Pathway

Accumulating evidence confirmed the important roles of NF-κB pathway in EMT-mediated radioresistance (2, 78). It has been shown that NF-κB pathway is involved in the regulation of EMT genes such as Twist, Slug and SIP1 in multiple cancers (22, 23). NF-κB activity can be induced by Notch signaling in tumor cells during injuries (79). Blockage of NF-κB activity by Notch inhibition could increase the sensitivity of NSCLC cells to radiotherapy (24). Similarly, inhibition of NF-κB signaling using its dominant-negative regulator A20, also significantly counteracted the formation of EMT and decreased the radioresistance of hepatocellular carcinoma (HCC) cells (25).

#### 2.1.6 IL-6/STAT3 Pathway

Activation of IL-6/STAT3 signaling has been shown to be instrumental in inducing EMT, which mediates progression and resistance to radiotherapy in many types of malignant cancers. Silence of STAT3 by siRNA blocked STAT3 activation and inhibited the mesenchymal phenotype of pancreatic cancer cells (80). Activation of STAT3 was involved in the regulation of PIM serine/threonine kinase (PIM2) expression, and targeting PIM2, STAT3 or PIM2-dependent cytokines could inhibit invasive and migratory properties of cancer cells possibly through suppression of Zeb1 (26). Blocking IL-6/STAT3 signaling by anti-IL-6 antibodies or STAT3 inhibitor (NSC74859) disturbed stellate cell-induced migration and expression of mesenchymal markers in pancreatic cancer cells (27). In addition, STAT3 knockdown could effectively suppress cancer stem cell-like properties, synergistically enhance the effects of radiotherapy, and significantly improve the survival of immunocompromised mice (81). These findings provide a valuable strategy to inhibit EMT and malignant phenotypes by blocking IL-6/STAT3 signaling.

#### 2.1.7 Other Relevant Molecular Mechanisms

**Figure 1:**
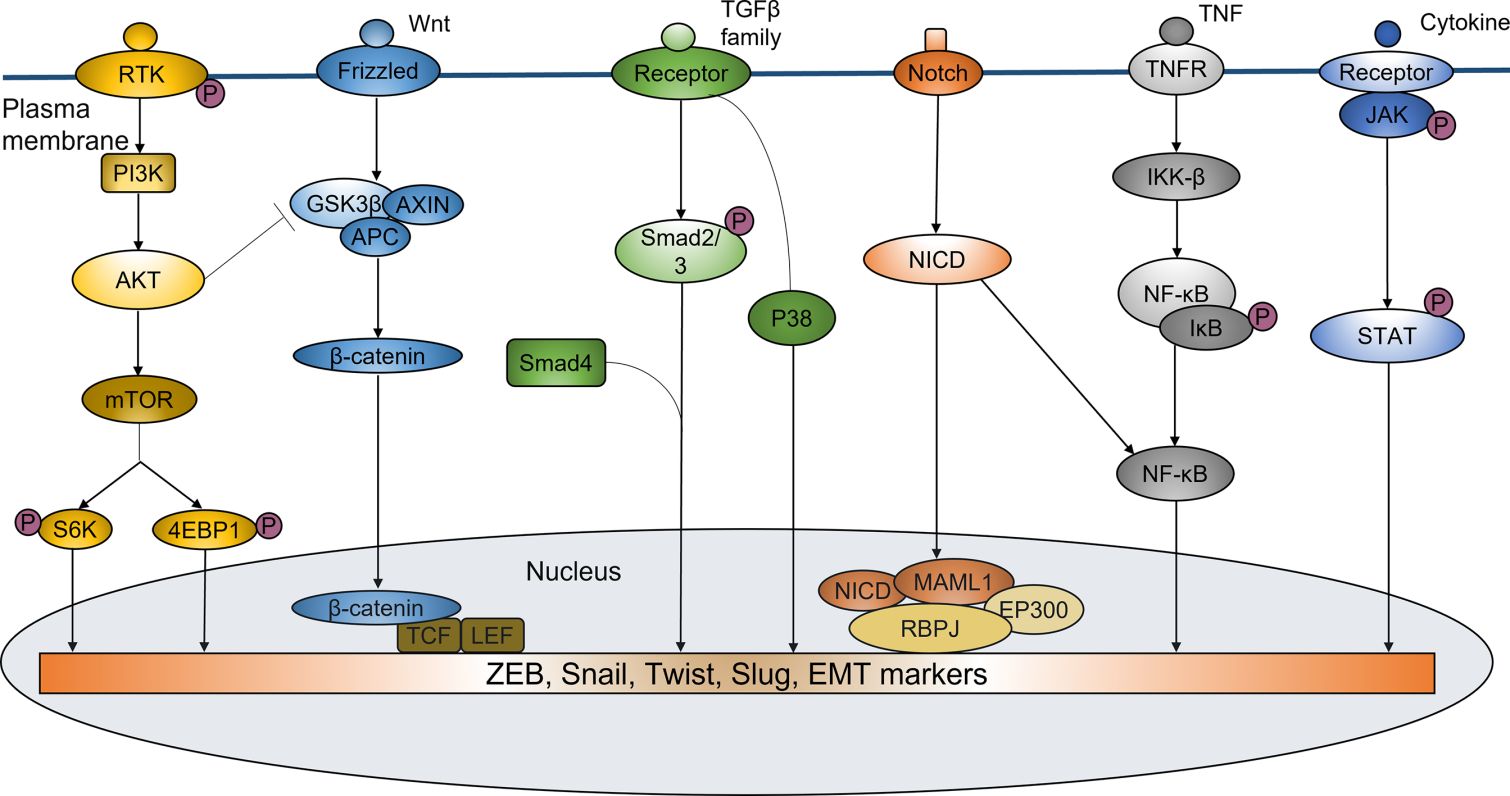
EMT-related signaling pathways in mediating radioresistance. EMT regulated by a variety of signaling pathways plays an important role in inducing ridioresistance of tumor cells. PI3K/Akt pathway participates in the regulation of Snail and Twist. PI3K/Akt activation can also increase phosphorylation, ubiquitination and degradation of GSK-3β, thus decreasing its degradation to Snail. Wnt/β-catenin signaling can promote the radioresistance by increasing nuclear translocation of β-catenin and transcriptional upregulation of EMT-related genes expression. TGF-β signaling can regulate the expression of EMT markers to induce EMT by Smad or non-Smad-dependent ways, thus enhancing the resistance of cancer cells to irradiation. Notch signaling can increase the expression of ZEB1, Slug, Snail, NF-κB and Vimentin to promote the EMT phenotype. NF-κB pathway has the important roles in EMT-mediated radioresistance. It is involved in the regulation of EMT genes such as Twist, Slug and SIP1. IL-6/JAK/STAT6 pathway can induce EMT by mediating ZEB1 and EMT-related markers, resulting in enhanced tumor resistance to radiotherapy.

Recent studies have identified sphingosine-1-phosphate receptor 1 (S1PR1) as a pro-oncogene that is highly expressed in a variety of malignancies. It plays a crucial role in promoting tumor metastasis and EMT process. The S1PR1 antagonist FTY720 can significantly inhibit EMT program by inactivating STAT3 signaling (82, 83), further modulating the resistance of cancer cells to therapy. Several studies have shown that S1PR1-mediated sustaining activation of STAT3 is of critical importance in proliferation and multidirectional differentiation of CSCs, which is closely associated with acquired radioresistance in a variety of malignancies such as pancreatic cancer and glioma (28–30). EMT-related signaling pathways in mediating radioresistance were shown in **Figure 1**.

### 2.2 Non-Coding RNAs in Radioresistance

Non-coding RNAs including microRNAs (miRNAs) and long non-coding RNAs (lncRNAs) have been considered as the important factors in EMT development and radioresistance by multiple regulatory mechanisms.

#### 2.2.1 MiRNAs

MiRNAs belong to small non-coding RNAs and act by inhibiting gene expression post-transcriptionally. One study demonstrated that ectopic miR-613 expression inhibited the proliferation and invasion, and impeded EMT phenotype by increasing expression of epithelial markers and decreasing expression of mesenchymal markers (84). Another study reported that miR30a decreased the invasion and migration of colorectal cancer cells, and its overexpression not only downregulated the expression levels of transmembrane-4-l-six-family-1 (TM4SF1), but inhibited VEGF expression and enhanced E-cadherin expression (85). MiR-3622a could inhibit the EMT by perturbing the expression of EMT effectors Zeb1 and Snail2 (86). All these findings suggest that multiple types of miRNAs exert tumor suppressive effects by counteracting EMT in cancer cells, and it may be a valuable tool for cancer therapy. In human colorectal cancer, miR-124 could sensitize cancer cells to ionizing radiation by directly targeting a new EMT inducer PRRX1 (31). MiR-145 negatively modulated EMT and radioresistance *via* regulating ZEB2 expression in human bladder cancer cells (32). Additionally, miR-205 has been verified to be an enhancer in radioresistance by promoting EMT in esophageal cell squamous carcinoma (ESCC) cells, which is enacted mechanistically through PTEN/PI3K/AKT signaling (33). Likewise, miR-624-3p interacting with CircVRK1 also modulated the radioresistance by this signaling-mediacted EMT (34). In ESCC cells, miR−301a is also validated for boosting radiosensitivity by suppressing Wnt1-dependent EMT (35).

#### 2.2.2 IncRNAs

Previous study has revealed that upregulation of IncRNA taurine gene 1 (TUG1) could elicit radioresistance by promoting EMT and targeting the miR-145/ZEB2 axis (32). Similarly, Lu et al. (36) reported that IncRNA-NEAT1 knockdown reversed the EMT phenotype by targeting miR-204/ZEB1 in NPC cells, suggesting that NEAT1 acts as an EMT inducer leading to radioresistance. Yang et al. (37) demonstrated that downregulation of IncRNA-UCA1 decreased the expression of EMT markers, such as MMP-9, ZEB1 and Vimentin, thereby improving radiosensitivity of colorectal cancer cells. As mentioned above, lncRNAs participate in the regulation of radiosensitivity of cancer cells by inducing or inhibiting EMT *via* different mechanisms.

### 2.3 Cancer Stem Cells

CSCs have the capacity of spheroid formation and self-renewal (87), which are randomly distributed in tumors (88), but are mainly localized in hypoxic, low pH and less nutritious ecological niches (89). CSCs play an important role in tumorigenesis, recurrence and metastasis (90–92). They share many features with regenerative stem cells, such as self-renewal and pluripotency (93), as well as their reversible quiescent state (94). However, there are numerous features specific to CSCs, including strong tumorigenic potential, proliferation, ALDH activity, and aberrant cell cycle (95). CSCs have been identified as a rare cell subpopulation within tumors (96, 97), representing 1% of the cells in most solid tumors (98).

EMT is interacted with CSCs in regulating tumorigenesis and development in a variety of cancers. On one hand, EMT works as an important manipulator in the acquisition of stem cell properties (38, 99–105). EMT-TF-inducted EMT program provoked the gain of tumor-initiating CSCs by regulating the expression of stem-cell markers in human breast cancer cells (38). CSCs is also verified to be closely linked with radioresistance (87, 106). In human lung cancer (LC) and NPC cells, the EGFR/PKM2 signaling enhanced the resistance of tumor cells to irradiation by inducing CSC-like features (107). Moreover, the radioresistant NSCLC cells not only showed the EMT phenotype but the enrichment of stemness markers including CD44 and CD133 (108). Therefore, EMT can drive radioresistance through promoting the transition of non-CSCs to CSCs. On the other hand, EMT is considered as a vital process in CSC plasticity (109). Evidence from the study of HCC cells proposed that CD29, one of the most studied CSC markers, was an inducer of EMT phenotype, and facilitated the radioresistance *via* modulating ILK-AKT-mTORC1 signaling (39). Another stemness factor CD44 is also involved in the regulation of radioresistance by driving EMT process *via* PI3K/AKT, Wnt/β-catenin or ERK pathways in prostate and pancreatic cancers (40, 41). These findings shed light on the interaction of EMT with CSCs in mediating radioresistance.

### 2.4 Tumor Microenvironment

**Figure 2:**
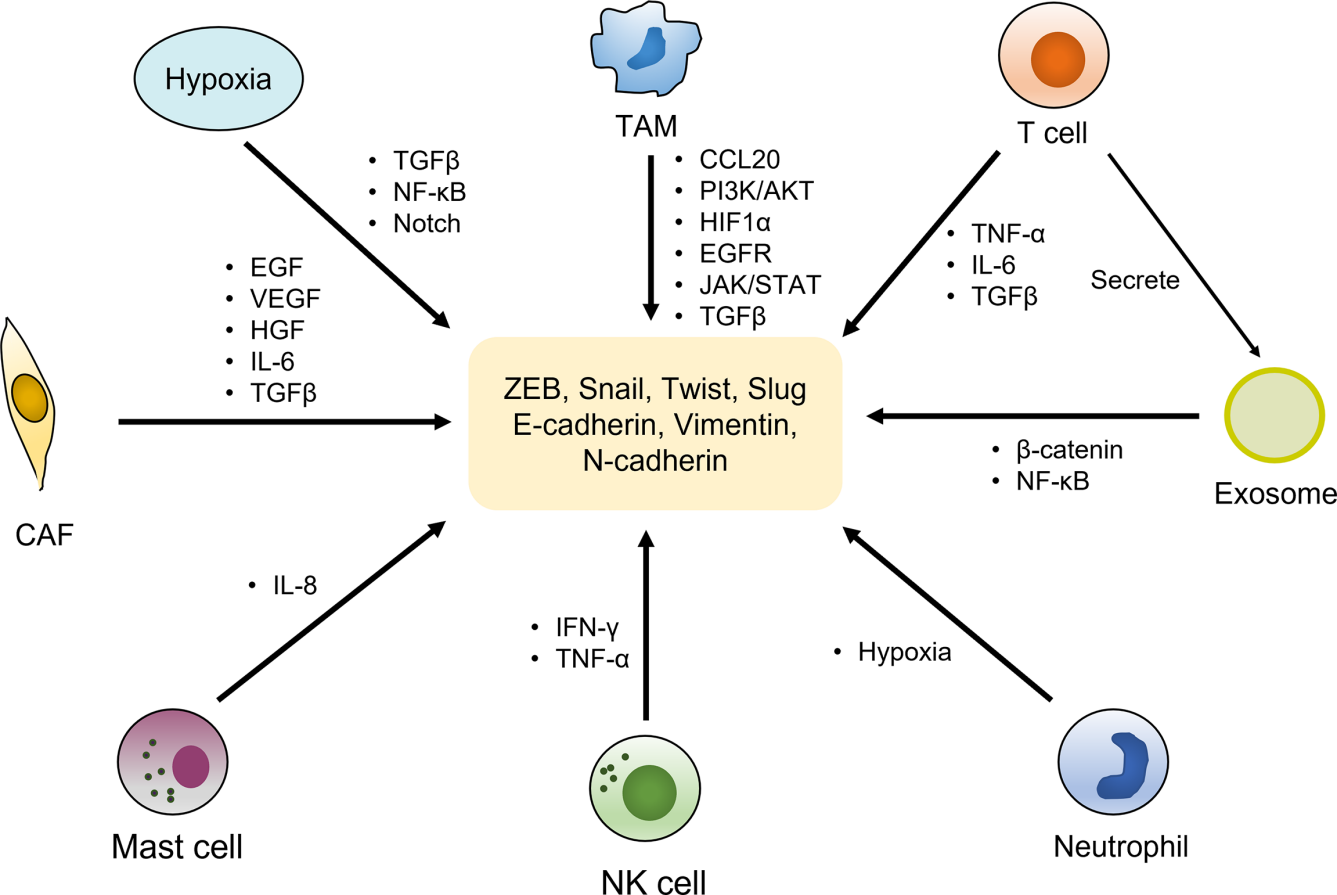
EMT activation by components of the tumor microenvironment. The tumor microenvironment is composed of cellular and non-cellular components. The cellular components mainly include immune cells (T cells, TAMs, neutrophil, NK cells, mast cells, etc.), cancer-associated fibroblasts (CAFs) and other types of cells, which activate the expression of various EMT transcriptional factors and markers by secreting cytokines and growth factors as well as regulating multiple signaling pathways, to induce EMT phenotype. Hypoxia environment as a non-cellular factor also exerts vital roles in trigger EMT by activating relevant pathways.

Tumor microenvironment (TME) has been confirmed to play a central role in EMT-induced radioresistance. It is composed of cellular and non-cellular components. The former mainly includes lymphocytes, macrophages, cancer-associated fibroblasts (CAFs), vascular endothelial cells and other types of cells, while the latter contains extracellular matrix (ECM), pH, oxygen partial pressure, and various metabolites. There is a complicated crosstalk between cancer cells and TME, interplaying with each other to facilitate tumor initiation and progression. Importantly, the alterations of TME factors strongly impinge on the development of EMT process (110). The roles of TEM in inducing EMT were displayed in **Figure 2**.

After radiotherapy, the number of local immunosuppressive cells such as tumor-associated macrophages (TAMs), myeloid-derived suppressor cells (MDSCs) and regulatory T cells in tumor tissue is relatively increased owing to their lower radiosensitivity compared to other lymphocyte subtypes (111–113). TAMs are a major component of tumor-infiltrating immunocytes and largely contribute to the immunosuppressive tumor microenvironment. M1-type macrophages efficiently phagocytose tumor cells and release pro-inflammatory cytokines that activate T cells and NK cells, whereas M2-type macrophages have immunosuppressive and TME remodeling functions. Studies showed that TAMs exert a crucial role in inducing EMT. Liu et al. (42) found that in pancreatic cancer, CCL20 released by M2-TAMs could facilitate invasion, metastasis and EMT of cancer cells mainly *via* regulating PI3K/AKT-ERK1/2 signaling pathway. COX-2(+) TAMs promoted the expression of MMP-9 and EMT phenotype in breast cancer cells by activating AKT signaling, thus promoting tumor cell invasion and metastasis (43). Also, M2 macrophages could release an array of cytokines to facilitate EMT *via* regulating multiple signaling pathways (44) such as HIF-1α (45), EGFR/ERK1/2 (46), Smad/Snail (47) and TGF-β (48, 49). Furthermore, TAMs could mediate EMT to promote the migration and invasion of cancer cells, which is associated with activation of JAK2/STAT3/miR-506-3p/FoxQ1 axis (50). Studies have reported that radiotherapy is involved in the regulation of many immune processes, such as antigen release and presentation, T lymphocyte initiation and activation, T cell recruitment and aggregation in tumors, and T lymphocyte recognition and killing of tumor cells (114). In addition to the roles against tumor cells, T cells can also mediate tumor progression. In inflammatory breast cancer (IBC), activated T cells could release soluble factors (TNF-α, IL-6, and TGF-β) to facilitate the expression of EMT-related genes, including FN1, VIM, TGM2 and ZEB1, thereby promoting EMT (51). Moreover, CD4(+) T cells in peritumor secrete large amount of IL-6 in clear cell renal carcinomas (ccRCC), which favors the alteration of tumor cell morphology as well as the acquisition of EMT and stemness phenotypes (52). It has been shown that radiotherapy can change the immune statement of the tumor microenvironment. After a local irradiation, the neutrophils can infiltrate into the tumor issue and alter irradiation response (115). Neutrophils function as a promoter in lung cancer progression. Neutrophils contribute to exacerbating hypoxia microenvironment and stabilizing Snail, which in turn enhances neutrophil homing and elicits partial EMT (53). Radiotherapy has been reported to enhance the cytotoxic effect of NK cells on tumor cells, as well as increase the ability of lymphocytes to translocate into tumors, while promoting cytokine production (116). However, NK cells were also found to enhance the malignancy of melanoma cells by inducing EMT-like changes, as evidenced with increased stemness and expression of EMT markers and morphologic switching, which depends on the release of IFN-γ and TNF-α. And the EMT phenotype assisted melanoma cells to escape from NK-cell killing by evaluating the NN-protective HLA-I expression or decreasing tumor-recognizing activating receptors on NK cells (54). Mast cells are one type of immune cells derived from hematopoietic stem/progenitor cells and are systemically present. Irradiation contributes to differentiation of bone marrow cells into mast cells, and affected the differentiation efficiency and function of mast cells (117, 118). Mast cells can release angiogenic-mediated VEGF by mediating MMP-9 in the tumor microenvironment after low-dose radiation therapy (119). One study demonstrated that mast cells could be recruited and activated by thyroid cancer cells in the TME, and release IL-8, which leads to EMT and tumor-initiating features of cancer cells (55). As for extracellular vesicles, radiation could stimulate the release of exosomes derived from T cells in human esophageal squamous cell carcinomas. Then exosomes induced the EMT phenotype of tumor cells *via* increasing the β-catenin expression and activating the NF-κB/snail pathway (56). Additionally, extracellular vesicle (EV)-mediated estrogen receptor-binding fragment-associated antigen 9 (EBAG9) from prostate cancer, has been proved to promote EMT by interacting with TM9SF1 to upregulate the expression of vimentin and Snail2 (120).

Radiotherapy can affect the expression of extracellular matrix components and ECM remodeling (113, 121). For instance, CAF activation following radiation leads to the secretion of numerous ECM proteins (113). Upregulation of an ECM protein SPARC could induce the occurrence of EMT in breast cancer cells, and leads to the TME reconstitution with increased infiltration of Tregs, mast cells, and MDSCs. SPARC-induced EMT was significantly correlated with MDSCs. The EMT program of cancer cells may be reverted by inhibition of suppressive function in MDSCs (122). Study found that fractionated irradiation had little effect on the morphology and capacity to contract collagen gels of CAFs, and it weakened growth and stimulated DNA-damage response of CAFs. Irradiated CAFs promoted survival of colorectal cancer (CRC) cells, and a metabolic switch favoring glutamine consumption *via* IGF-1 receptor activation (123). CAFs contribute to the gain of EMT phenotype of tumor cells, endowing them with enhanced therapeutic resistance and metastatic property (124). It has been confirmed that CAFs can induce EMT through paracrine TGF-β to provoke the aggressive phenotype of cancer cells (57). Additionally, CAFs can produce a lot of cytokines and growth factors, such as IL-6, EGF, VEGF and HGF, to mediate EMT (57–60). Furthermore, co-culture of esophageal cancer cells with irradiated fibroblasts provoked the cancer cells undergoing the EMT program, and increased the expression of HDGF, which are the identified mechanisms of tumor radioresistance (125).

The hypoxic microenvironment has been identified as one of the most important factors in eliciting EMT that mainly acts through hypoxia inducible factors (HIFs). Under hypoxic circumstances, the TGF-β, NF-κB and Notch signaling pathways that directly trigger EMT are activated (61, 62). HIF-1α is implicated in the regulation of transcription factors such as Zebl, Snail, Twist, and promote the EMT process (63). Theys et al. (1) uncovered that upon hypoxia, TGF-β addition or EGFRvIII expression, promoted the breast cancer and LC cells to acquire EMT-like phenotype, accompanied by the decreased expression of epithelial markers such as E-cadherin and increased expression of mesenchymal markers such as vimentin and N-cadherin. Intriguingly, the treatment of reoxygenation could reverse the E-cadherin expression and the mesenchymal phenotype, and mesenchymal conversion and E-cadherin loss were associated with resistance of tumor cells to irradiation. The development of EMT is a comprehensive network involving transcription, post-transcriptional regulation and cytokines from the surrounding environment. TME plays an important role in EMT and is one of the key factors in EMT-mediated radioresistance.

## 3 Irradiation-Mediated EMT Program

There is an interactive network between EMT and irradiation. The processes of EMT induces radioresistance of tumors, and irradiation further enhances EMT, providing a positive feedback loop to increase the radioresistance. After several hour’s irradiation to cancer cells with X-rays, the migration and invasion capacities of these cells were increased, paralleled by a decrease of E-cadherin expression and an increase of Vimentin and Smad3 expression (126). Tsukamoto H et al. (127) also found that irradiation facilitated the migration and invasion of endometrial cancer cells by inducing the development of EMT *in vitro*.

In tumorigenesis and development, a variety of signaling pathways are significantly activated, such as MAPK/ERK, TGFβ, HIF-1, Wnt and Notch pathways, and irradiation will further activate these pathways to induce the tumor malignant phenotypes such as EMT, leading to the occurrence of radioresistance. Irradiation can increase the release of the reactive oxygen species (ROS), resulting in the activation of signaling pathways and alterations in the tumor microenvironment. ROS has been reported to be involved in irradiation-induced EMT by regulation of multiple EMT markers and transcriptional factors (128). Evaluated levels of ROS may contribute to activating NF-κB signaling, which increases IL-6 production by cancer cells and CAFs, thus facilitating the EMT program by regulating the expression of N-cadherin, E-cadherin, vimentin, Twist and Snail (129). In addition, irradiation could promote the upregulation of Snail *via* activating the ERK signaling and inhibiting glycogen synthase kinase 3β (GSK3β) (130, 131). Elimination of ROS using a ROS scavenger N-acetyl-L-cysteine (LNAC), suppressed the expression of vimentin, indicative of a vital role for ROS in irradiation-induced EMT (132).

P38 mitogen-activated protein kinase (p38MAPK) pathway was activated after irradiation, which contributes to the occurrence of EMT and invasion through increased expression of Snail (133). HIF-1 was also implicated in irradiation-induced EMT. Irradiation could increase the protein stability of HIF-1α under hypoxia, and then HIF-1α translocated to the nucleus, dimerized with HIF-1β, and regulated the gene expression of key EMT transcriptional factors, thereby inducing EMT, migration and invasion (134). Furthermore, irradiation could activate Wnt signaling pathway by mediating Wnt ligand expression, increasing β-catenin stabilization and transcriptional activities (70, 128, 135), which strengthens the expression of Snail to trigger EMT (71). Exposure to irradiation can promote the activation of the TGF-β signaling, leading to the EMT in many types of cancer cells (9, 136, 137). Disturbance of TGF-β pathway could increase the radiosensitivity *via* repressing EMT (10). Moreover, another study discovered that EMT induced by irradiation through TGF-β pathway was not limited to the radiation dose range, and both low- and high-level radiation could contribute to the induction of EMT (138). Likewise, Zhou Y C et al. (139) irradiated six cancer cells lines with 2Gy dose, and they found that the irradiated cancer cells presented enhanced invasion and metastatic abilities, which is associated with irradiated-induced EMT process by activating TGF-β signaling to regulate the expression of epithelial and mesenchymal markers. Li T et al. (140) demonstrated that irradiation strengthened the expression of TMPRSS4, and subsequently weakened the expression of E-cadherin, which triggers EMT to enhance the invasion and metastasis of cancer cells. Additionally, NF-κB pathway was activated by irradiation in A549 cells, involved in the induction of EMT (141). NF-κB could elicit STAT3 activation by transcriptional upregulation of HER2 expression after exposure to ionizing radiation, thus resulting in EMT-mediated radioresistance in breast cancer stem cells (142–144). Besides, evidence from another study of breast cancer uncovered that Notch signaling was highly activated in fractionally irradiated cancer cells, accompanied with the alteration of EMT phenotype. Afterward, it revealed that Notch provoked the EMT by regulating IL-6/JAK/STAT6 signaling pathway in response to fractionated-radiation (145).

Taken together, multiple signaling pathways such as Wnt, TGF-β, NF-κB, Notch and HIF-1 pathways play a crucial role in the occurrence of radiotherapy-induced EMT. Meanwhile, the alterations in TME induced by radiotherapy such as increase of ROS may acts as a stimulator to involve in the activation of signaling pathways.

## 4 Perspectives on Cancer Treatment

From the primary cancer to adjacent and distant organs, EMT confers the potential of tumor invasion and metastasis, as well as the capacity of inducing therapeutic resistance. Therefore, targeting EMT by attenuating the mesenchymal/CSC phenotypes, inhibiting EMT-related signaling pathways, and perturbing tumorigenic TME, will be considered as promising therapeutic strategies for tumor treatment. The studies of these therapeutic strategies are summarized in **Table 2**.

**Table 2:** Novel strategies targeting EMT-induced radioresistance.

Therapy	Target	Function	Reference
Ionophore salinomycin	CSC	Inhibition CSC phenotype	(146, 147)
Ruxolitinib	JAK1/2	Block the STAT3-mediated transcription of Zeb1 and Snai1, and disturb the CSC phenotype	(148)
BEZ235	PI3K/Akt/mTOR	Decrease the expression of EMT/CSC markers	(17)
Simvastatin	PI3K/Akt	Suppress PTEN-PI3K/Akt pathway and promote the radiosensitivity	(149)
PF-05212384	PI3K/mTOR	Inhibit PI3K and mTOR and increase radiosensitivity	(150)
Everolimus	Akt/mTOR	Inhibit Akt/mTOR pathway	(151–153)
Nimotuzumab	EGFR	Weaken radioresistance by inhibiting EGFR signaling pathway and DNA repair	(154)
Cetuximab	EGFR	Increase the radiosensitivity	(155)
GSI (RO4929097)	Notch	Suppress Notch signaling	(156)
Tangeretin	Notch	Enhance the radiosensitivity and counteract irradiation-induced EMT	(157)
Rhamnetin and cirsiliol	Notch	Reverse EMT phenotype and improve radiosensitivity	(24)
FH535	β-Catenin/Tcf	Inhibit the activation of Wnt/β-catenin pathway and increasing E-cadherin expression	(13)
Sunitinib	Hypoxia	Reduce tumor hypoxia and angiogenesis, and radiosensitize cancer stem-like cells	(158)
Paclitaxel	Hypoxia	Overcome HIF-1α-induced radioresistance	(159)
Sorafeinib	Hypoxia	Suppress HIF-1α expression	(160)
Bortezomib	Hypoxia	Suppress HIF-1α expression	(161)
Albumin–MnO2 nanoparticles	Hypoxia	Improve hypoxic environment, and strengthen the radiosensitivity	(162)
Acriflavine	HIF1	Suppress HIF1 dimerization and transcriptional activity	(163)
YC−1	HIF1	Inhibit HIF1 and enhance radiosensitivity	(164)

Previous studies have verified that CSC traits are associated with mesenchymal phenotype, metastatic capacity and therapy resistance. Targeting the CSC phenotype was conducive to the acquisition of considerable therapeutic benefit (165). The ionophore salinomycin is confirmed to be 100-fold more active against CSCs than traditional chemotherapy agent paclitaxel in breast cancer (146). Notably, the treatment of tumors with salinomycin significantly reduced the tumor size compared with the control group (147).

Given the important role of signaling pathways in mediating EMT, it is promising to acquire radiosensitization by combining the inhibitors targeted against these pathways with radiotherapy. Oncomodulin M (OSM) is a member of the IL6 cytokine family, which has been identified as an important driver of mesenchymal and CSC phenotypes. Inhibition of JAK1/2 by ruxolitinib blocked the STAT3-mediated transcription of OSM receptors Zeb1 and Snai1 and disturbed the emergence of CSC phenotype in pancreatic ductal adenocarcinoma cells (148). Inhibition of the PI3K/Akt pathway has been proven to be an effective way to increase the sensitivity of cancer cells to radiotherapy. Chang et al. reported that application of PI3K/Akt/mTOR pathway inhibitor (BEZ235) in combined with RT could significantly weaken radioresistance by decreasing the expression of EMT/CSC markers (17). Simvastatin, a conventional drug that is utilized for cardiovascular diseases, has been reported to promote the radiosensitivity of esophageal cancer cells by suppressing PTEN-PI3K/Akt pathway (149). In preclinical models, a dual PI3K/mTOR inhibitor PF-05212384 effectively inhibited PI3K and mTOR, leading to increased sensitivity of HNSCC to radiation (150). Another Akt/mTOR inhibitor Everolimus (also known as Rad001) are currently undergoing clinical trials in combination with radiotherapy in a variety of tumors, and may become a useful method of radiosensitization (151–153). Additionally, Nimotuzumab, an anti-EGFR monoclonal antibody, exerts effective roles in improving the radioresistance of esophageal cancer KYSE-150R cells by inhibiting EGFR signaling pathway and DNA repair (154). Also, Cetuximab, another anti-EGFR pathway antibody, displays potential antitumor effect in combination with radiotherapy, and obtained a survival benefit of 10% in patients with head and neck squamous cell carcinoma (HNSCC) in a phase 3 clinical trial (155). Several types of Notch pathway inhibitors are in clinical development, especially γ−secretase inhibitors (GSIs), which disturb the proteolytic cleavage of Notch receptors and block the release of the active intracellular domain (NICD). In multiple preclinical models, these inhibitors have displayed significant anti-tumor activity. Noteworthily, GSI (RO4929097) in combination with RT has entered clinical trials in patients with gliomas (NTC01119599) or brain metastases (NCT01217411) (156). In gastric cancer (GC), tangeretin that acts as a Notch-1 inhibitor could enhance the radiosensitivity and counteract irradiation-induced EMT both *in vitro* and *in vivo* (157). Blockage of Notch signaling by rhamnetin and cirsiliol could result in the reversal of EMT phenotype and improvement of radiosensitivity in NSCLC (24). These findings implied that inhibiting EMT by blockage of the Notch pathway may be an effective means to reduce EMT-mediated radioresistance. Wnt pathway plays a protective role for cancer cells in response to IR, therefore, targeting the Wnt signaling may also be important for enhancing radiosensitization. FH535, a β-Catenin/Tcf inhibitor that can inhibit the activation of Wnt/β-catenin pathway, is known as a potential radiosensitizer. It could effectively suppress EMT to reverse the radioresistance of KYSE-150R cells by increasing E-cadherin expression (13). Other small molecule inhibitors have been used in preclinical trials or clinical trials and shown good efficacy in blocking the Wnt signaling pathway (156, 166–168). Currently, several HIF-1-targeting agents have been applied in clinical practice, such as sunitinib (158), paclitaxel (159), solafeinib (160) and bortezomib (161). These agents, as antineoplastic chemotherapy or targeted treatment, can be combined with radiotherapy to play the role of radiosensitization. Furthermore, with the rapid development of nanotechnology, the method generating oxygen in hypoxic tumor regions has been accomplished by intratumoral injection of albumin–MnO2 nanoparticles, which will conduce to the improvement of hypoxic environment and strengthen the radiosensitivity (162). Acriflavine has been revealed that could suppress HIF1 dimerization and transcriptional regulation *via* directly binding to the HIF1α subunit (163). The application of YC−1, a HIF1 inhibitor, also displayed the treatment benefits when administered after irradiation (164).

In addition to the agents mentioned above, there are several old drugs that also have potential radiosensitizing effects. The anti-diabetic drug metformin can suppress the EMT program and stemness by regulating transcriptional reprogramming and inhibiting ZEB1, TWIST1 and SLUG (169, 170), and strengthens the sensitivity of cancer cells to irradiation (171). Berberine could reduce the expression of vimentin and evaluate the level of E-cadherin, thereby perturbing the TGF-β-induced EMT and sensitizing nasopharyngeal carcinoma cells to radiation (10).

Although many agents exibited effective roles in radiosensitization, they are rarely applied in clinical practice as radiosensitizers. We consider the possible reasons for blocking the transformation of these agents as follows. First, the efficacy of many agents is verified *in vitro* experiments or in preclinical animal experiments, however, no good therapeutic effects is observed in clinical trials. Second, the limitation of transformation may be partly attributed to safety of the agents. Combined application of the agents with radiotherapy may amplify adverse reactions, even leading to some serious complications, which is an important reason for restricting the clinical application of many agents. Third, the molecular mechanisms involved in EMT are extremely complex, and these factors do not operate isolation, but functions as a coordinated signaling network, ultimately leading to the output of EMT program. Therefore, a comprehensive understanding aiming to the key nodes in this network is needed for the development of EMT-targeting radiosensitizers, which increases the difficulty of drug transformation. Last, drug delivery into cancer cells is also an important factor that needs to be solved urgently. Drug transport into malignant tumors through blood relies on a variety of factors, and is affected by tumor vascular system and tissue characteristics. Moreover, irradiation has a significant impact on the revascularization of tumor tissues, so how to increase the drug concentration at the tumor site may also be a factor that limits the radiosensitivity. Currently, the application of microcarriers based on nanoparticles or liposomes may provide a potential method to improve the tumor-killing effect and increase the sensitivity of radiotherapy.

## 5 Conclusions

**Figure 3:**
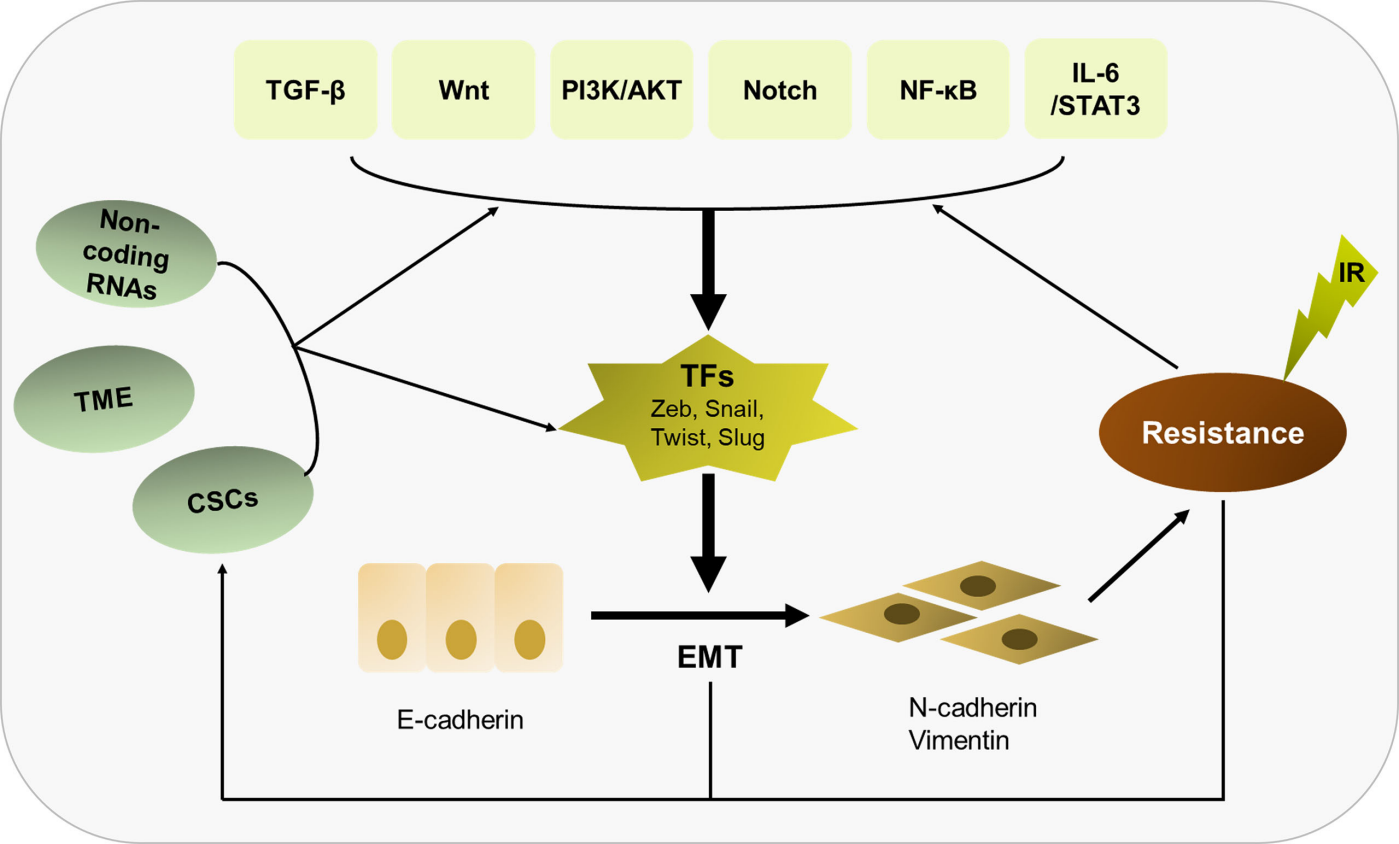
Overview of the relationship of EMT with radioresistance and their crosslinked mechanisms. EMT plays a central role in eliciting radioresistance. Multiple molecular mechanisms are involved in EMT-induced radioresistance, including TGF-β, Wnt, PI3K/AKT, Notch, NF-κB, IL-6/STAT3, non-coding RNAs, CSCs and tumor microenvironment. Besides, there is a vicious circle between EMT and irradiation. Irradiation can further enhance the EMT program, thus strengthening radioresistance.

In summary, radioresistance is still regarded as a major challenge in current tumor treatment, and EMT as an important inducer of radioresistance has attracted large amount of attention. In this review, we hold the opinion that EMT is not only the cause to induce radioresistance, but the result of irradiation acting on cancer cells and TMEs, thus forming an infinitely amplified positive feedback network to further increase the resistance of tumor cells to radiotherapy (**Figure 3**). We must comprehensively recognize that EMT plays a central role in eliciting resistance of tumor cells to radiotherapy, involved in multiple molecular mechanisms including signaling pathways, non-coding RNAs, CSCs and tumor microenvironment. The fact that there is a vicious circle between EMT and irradiation needs to be better understood. At the present, there are an array of agents in preclinical or clinical trials that work on EMT or its related signal pathways. Based on continuous progression on molecular medicine and novel biotechnology, it holds promise in overcoming radioresistance and improving the prognosis of cancer patients.

## Author Contributions

JZ and LQ conceived the framework of manuscript. LQ and YC were responsible for writing the manuscript. NL, YL, JX and XW conducted the figure and tables. FL, GD, and FC performed the revision of the manuscript. All authors have read and approved the final manuscript.

## Funding

This project was funded by the National Natural Science Foundation of China (grant no. 81672974) and the Natural Science Foundation of Shandong Province (grant no. ZR2021QH356 and no. ZR2021LSW023). The funders are the corresponding author and first author of this paper.

## Conflict of Interest

The authors declare that the research was conducted in the absence of any commercial or financial relationships that could be construed as a potential conflict of interest.

## Publisher’s Note

All claims expressed in this article are solely those of the authors and do not necessarily represent those of their affiliated organizations, or those of the publisher, the editors and the reviewers. Any product that may be evaluated in this article, or claim that may be made by its manufacturer, is not guaranteed or endorsed by the publisher.

## References

[1] Theys J Jutten B Habets R Paesmans K Groot AJ Lambin P. E-Cadherin Loss Associated With EMT Promotes Radioresistance in Human Tumor Cells. Radiother Oncol: J Eur Soc Ther Radiol Oncol (2011) 99(3):392–7. doi: 10.1016/j.radonc.2011.05.044 PMC4948667 21680037

[2] Nantajit D Lin D Li JJ. The Network of Epithelial-Mesenchymal Transition: Potential New Targets for Tumor Resistance. J Cancer Res Clin Oncol (2015) 141(10):1697–713. doi: 10.1007/s00432-014-1840-y PMC4382462 25270087

[3] Karnevi E Rosendahl AH Hilmersson KS Saleem MA Andersson R. Impact by Pancreatic Stellate Cells on Epithelial-Mesenchymal Transition and Pancreatic Cancer Cell Invasion: Adding a Third Dimension *In Vitro* . Exp Cell Res (2016) 346(2):206–15. doi: 10.1016/j.yexcr.2016.07.017 27443257

[4] Zhou S Zhang M Zhou C Wang W Yang H Ye W. The Role of Epithelial-Mesenchymal Transition in Regulating Radioresistance. Crit Rev Oncol/Hematol (2020) 150(2020):102961. doi: 10.1016/j.critrevonc.2020.102961 32361589

[5] Puisieux A Brabletz T Caramel J. Oncogenic Roles of EMT-Inducing Transcription Factors. Nat Cell Biol (2014) 16(6):488–94. doi: 10.1038/ncb2976 24875735

[6] Peinado H Olmeda D Cano A. Snail, Zeb and bHLH Factors in Tumour Progression: An Alliance Against the Epithelial Phenotype? Nat Rev Cancer (2007) 7(6):415–28. doi: 10.1038/nrc2131 17508028

[7] Wang Y Shi J Chai K Ying X Zhou BP. The Role of Snail in EMT and Tumorigenesis. Curr Cancer Drug Targets (2013) 13(9):963–72. doi: 10.2174/15680096113136660102 PMC4004763 24168186

[8] Al-Assar O Demiciorglu F Lunardi S Gaspar-Carvalho MM McKenna WG Muschel RM. Contextual Regulation of Pancreatic Cancer Stem Cell Phenotype and Radioresistance by Pancreatic Stellate Cells. Radiother Oncol: J Eur Soc Ther Radiol Oncol (2014) 111(2):243–51. doi: 10.1016/j.radonc.2014.03.014 24780634

[9] Konge J Leteurtre F Goislard M Biard D Morel-Altmeyer S Vaurijoux A. Breast Cancer Stem Cell-Like Cells Generated During Tgfβ-Induced EMT are Radioresistant. Oncotarget (2018) 9(34):23519–31. doi: 10.18632/oncotarget.25240 PMC5955125 29805752

[10] Wang J Kang M Wen Q Qin YT Wei ZX Xiao JJ. Berberine Sensitizes Nasopharyngeal Carcinoma Cells to Radiation Through Inhibition of Sp1 and EMT. Oncol Rep (2017) 37(4):2425–32. doi: 10.3892/or.2017.5499 28350122

[11] Yang XZ Chen XM Zeng LS Deng J Ma L Jin C. Rab1A Promotes Cancer Metastasis and Radioresistance Through Activating GSK-3β/Wnt/β-Catenin Signaling in Nasopharyngeal Carcinoma. Aging (2020) 12(20):20380–95. doi: 10.18632/aging.103829 PMC7655155 33068388

[12] Cojoc M Peitzsch C Kurth I Trautmann F Kunz-Schughart LA Telegeev GD. Aldehyde Dehydrogenase Is Regulated by β-Catenin/TCF and Promotes Radioresistance in Prostate Cancer Progenitor Cells. Cancer Res (2015) 75(7):1482–94. doi: 10.1158/0008-5472.CAN-14-1924 25670168

[13] Su H Jin X Zhang X Zhao L Lin B Li L. FH535 Increases the Radiosensitivity and Reverses Epithelial-to-Mesenchymal Transition of Radioresistant Esophageal Cancer Cell Line KYSE-150r. J Trans Med (2015) 13:104. doi: 10.1186/s12967-015-0464-6 PMC4384308 25888911

[14] Luo M Wu C Guo E Peng S Zhang L Sun W. FOXO3a Knockdown Promotes Radioresistance in Nasopharyngeal Carcinoma by Inducing Epithelial-Mesenchymal Transition and the Wnt/β-Catenin Signaling Pathway. Cancer Lett (2019) 455:26–35. doi: 10.1016/j.canlet.2019.04.019 31022422

[15] Lee YJ Han HJ. Troglitazone Ameliorates High Glucose-Induced EMT and Dysfunction of SGLTs Through PI3K/Akt, GSK-3β, Snail1, and β-Catenin in Renal Proximal Tubule Cells. Am J Physiol Renal Physiol (2010) 298(5):F1263–75. doi: 10.1152/ajprenal.00475.2009 20015942

[16] Xu W Yang Z Lu N. A New Role for the PI3K/Akt Signaling Pathway in the Epithelial-Mesenchymal Transition. Cell Adhesion Migration (2015) 9(4):317–24. doi: 10.1080/19336918.2015.1016686 PMC4594353 26241004

[17] Chang L Graham PH Hao J Ni J Bucci J Cozzi PJ. Acquisition of Epithelial-Mesenchymal Transition and Cancer Stem Cell Phenotypes is Associated With Activation of the PI3K/Akt/mTOR Pathway in Prostate Cancer Radioresistance. Cell Death Dis (2013) 4(10):e875. doi: 10.1038/cddis.2013.407 24157869 PMC3920940

[18] Ni J Cozzi P Hao J Beretov J Chang L Duan W. Epithelial Cell Adhesion Molecule (EpCAM) is Associated With Prostate Cancer Metastasis and Chemo/Radioresistance *via* the PI3K/Akt/mTOR Signaling Pathway. Int J Biochem Cell Biol (2013) 45(12):2736–48. doi: 10.1016/j.biocel.2013.09.008 24076216

[19] Wang Z Li Y Kong D Banerjee S Ahmad A Azmi AS. Acquisition of Epithelial-Mesenchymal Transition Phenotype of Gemcitabine-Resistant Pancreatic Cancer Cells is Linked With Activation of the Notch Signaling Pathway. Cancer Res (2009) 69(6):2400–7. doi: 10.1158/0008-5472.CAN-08-4312 PMC2657919 19276344

[20] Yu S Zhang R Liu F Hu H Yu S Wang H. Down-Regulation of Notch Signaling by a γ-Secretase Inhibitor Enhances the Radiosensitivity of Nasopharyngeal Carcinoma Cells. Oncol Rep (2011) 26(5):1323–8. doi: 10.3892/or.2011.1402 21805038

[21] Wang J Wakeman TP Lathia JD Hjelmeland AB Wang XF White RR. Notch Promotes Radioresistance of Glioma Stem Cells. Stem Cells (Dayton Ohio) (2010) 28(1):17–28. doi: 10.1002/stem.261 PMC2825687 19921751

[22] Pires BR Mencalha AL Ferreira GM de Souza WF Morgado-Díaz JA Maia AM. NF-kappaB Is Involved in the Regulation of EMT Genes in Breast Cancer Cells. PloS One (2017) 12(1):e0169622. doi: 10.1371/journal.pone.0169622 28107418 PMC5249109

[23] Huber MA Beug H Wirth T. Epithelial-Mesenchymal Transition: NF-kappaB Takes Center Stage. Cell Cycle (Georgetown Tex) (2004) 3(12):1477–80. doi: 10.4161/cc.3.12.1280 15539952

[24] Kang J Kim E Kim W Seong KM Youn H Kim JW. Rhamnetin and Cirsiliol Induce Radiosensitization and Inhibition of Epithelial-Mesenchymal Transition (EMT) by miR-34a-Mediated Suppression of Notch-1 Expression in non-Small Cell Lung Cancer Cell Lines. J Biol Chem (2013) 288(38):27343–57. doi: 10.1074/jbc.M113.490482 PMC3779729 23902763

[25] Liu R Zhao D Zhang X Han S Yang Y Ma J. A20 Enhances the Radiosensitivity of Hepatocellular Carcinoma Cells to 60Co-γ Ionizing Radiation. Oncotarget (2017) 8(54):93103–16. doi: 10.18632/oncotarget.21860 PMC5696247 29190981

[26] Uddin N Kim RK Yoo KC Kim YH Cui YH Kim IG. Persistent Activation of STAT3 by PIM2-Driven Positive Feedback Loop for Epithelial-Mesenchymal Transition in Breast Cancer. Cancer Sci (2015) 106(6):718–25. doi: 10.1111/cas.12668 PMC4471798 25854938

[27] Hamada S Masamune A Yoshida N Takikawa T Shimosegawa T. IL-6/STAT3 Plays a Regulatory Role in the Interaction Between Pancreatic Stellate Cells and Cancer Cells. Digest Dis Sci (2016) 61(6):1561–71. doi: 10.1007/s10620-015-4001-5 26738736

[28] Marvaso G Barone A Amodio N Raimondi L Agosti V Altomare E. Sphingosine Analog Fingolimod (FTY720) Increases Radiation Sensitivity of Human Breast Cancer Cells *In Vitro* . Cancer Biol Ther (2014) 15(6):797–805. doi: 10.4161/cbt.28556 24657936 PMC4049795

[29] Masliantsev K Pinel B Balbous A Guichet PO Tachon G Milin S. Impact of STAT3 Phosphorylation in Glioblastoma Stem Cells Radiosensitization and Patient Outcome. Oncotarget (2017) 9(3):3968–79. doi: 10.18632/oncotarget.23374 PMC5790515 29423098

[30] Wu X Tang W Marquez RT Li K Highfill CA He F. Overcoming Chemo/Radio-Resistance of Pancreatic Cancer by Inhibiting STAT3 Signaling. Oncotarget (2016) 7(10):11708–23. doi: 10.18632/oncotarget.7336 PMC4905505 26887043

[31] Zhang Y Zheng L Huang J Gao F Lin X He L. MiR-124 Radiosensitizes Human Colorectal Cancer Cells by Targeting PRRX1. PloS One (2014) 9(4):e93917. doi: 10.1371/journal.pone.0093917 24705396 PMC3976353

[32] Tan J Qiu K Li M Liang Y. Double-Negative Feedback Loop Between Long non-Coding RNA TUG1 and miR-145 Promotes Epithelial to Mesenchymal Transition and Radioresistance in Human Bladder Cancer Cells. FEBS Lett (2015) 589(20 Pt B):3175–81. doi: 10.1016/j.febslet.2015.08.020 26318860

[33] Pan F Mao H Bu F Tong X Li J Zhang S. Sp1-Mediated Transcriptional Activation of miR-205 Promotes Radioresistance in Esophageal Squamous Cell Carcinoma. Oncotarget (2017) 8(4):5735–52. doi: 10.18632/oncotarget.13902 PMC5351585 27974696

[34] He Y Mingyan E Wang C Liu G Shi M Liu S. CircVRK1 Regulates Tumor Progression and Radioresistance in Esophageal Squamous Cell Carcinoma by Regulating miR-624-3p/PTEN/PI3K/AKT Signaling Pathway. Int J Biol Macromol (2019) 125:116–23. doi: 10.1016/j.ijbiomac.2018.11.273 30508543

[35] Su H Wu Y Fang Y Shen L Fei Z Xie C. MicroRNA-301a Targets WNT1 to Suppress Cell Proliferation and Migration and Enhance Radiosensitivity in Esophageal Cancer Cells. Oncol Rep (2019) 41(1):599–607. doi: 10.3892/or.2018.6799 30365079

[36] Lu Y Li T Wei G Liu L Chen Q Xu L. The Long non-Coding RNA NEAT1 Regulates Epithelial to Mesenchymal Transition and Radioresistance in Through miR-204/ZEB1 Axis in Nasopharyngeal Carcinoma. Tumour Biol: J Int Soc Oncodevelopmental Biol Med (2016) 37(9):11733–41. doi: 10.1007/s13277-015-4773-4 27020592

[37] Yang X Liu W Xu X Zhu J Wu Y Zhao K. Downregulation of Long non−Coding RNA UCA1 Enhances the Radiosensitivity and Inhibits Migration *via* Suppression of Epithelial−Mesenchymal Transition in Colorectal Cancer Cells. Oncol Rep (2018) 40(3):1554–64. doi: 10.3892/or.2018.6573 30015983

[38] Mani SA Guo W Liao MJ Eaton EN Ayyanan A Zhou AY. The Epithelial-Mesenchymal Transition Generates Cells With Properties of Stem Cells. Cell (2008) 133(4):704–15. doi: 10.1016/j.cell.2008.03.027 PMC2728032 18485877

[39] Jiang X Wang J Zhang K Tang S Ren C Chen Y. The Role of CD29-ILK-Akt Signaling-Mediated Epithelial-Mesenchymal Transition of Liver Epithelial Cells and Chemoresistance and Radioresistance in Hepatocellular Carcinoma Cells. Med Oncol (Northwood London England) (2015) 32(5):141. doi: 10.1007/s12032-015-0595-x 25805567

[40] Ni J Cozzi PJ Hao JL Beretov J Chang L Duan W. CD44 Variant 6 is Associated With Prostate Cancer Metastasis and Chemo-/Radioresistance. Prostate (2014) 74(6):602–17. doi: 10.1002/pros.22775 24615685

[41] Tsubouchi K Minami K Hayashi N Yokoyama Y Mori S Yamamoto H. The CD44 Standard Isoform Contributes to Radioresistance of Pancreatic Cancer Cells. J Radiat Res (2017) 58(6):816–26. doi: 10.1093/jrr/rrx033 PMC5710530 29106581

[42] Liu B Jia Y Ma J Wu S Jiang H Cao Y. Tumor-Associated Macrophage-Derived CCL20 Enhances the Growth and Metastasis of Pancreatic Cancer. Acta Biochim Biophys Sin (2016) 48(12):1067–74. doi: 10.1093/abbs/gmw101 27797715

[43] Maolake A Izumi K Shigehara K Natsagdorj A Iwamoto H Kadomoto S. Tumor-Associated Macrophages Promote Prostate Cancer Migration Through Activation of the CCL22-CCR4 Axis. Oncotarget (2017) 8(6):9739–51. doi: 10.18632/oncotarget.14185 PMC5354767 28039457

[44] Chen Y Tan W Wang C. Tumor-Associated Macrophage-Derived Cytokines Enhance Cancer Stem-Like Characteristics Through Epithelial-Mesenchymal Transition. OncoTargets Ther (2018) 11:3817–26. doi: 10.2147/OTT.S168317 PMC6038883 30013362

[45] Zhang J Zhang Q Lou Y Fu Q Chen Q Wei T. Hypoxia-Inducible Factor-1α/Interleukin-1β Signaling Enhances Hepatoma Epithelial-Mesenchymal Transition Through Macrophages in a Hypoxic-Inflammatory Microenvironment. Hepatol (Baltimore Md) (2018) 67(5):1872–89. doi: 10.1002/hep.29681 29171040

[46] Gao L Zhang W Zhong WQ Liu ZJ Li HM Yu ZL. Tumor Associated Macrophages Induce Epithelial to Mesenchymal Transition *via* the EGFR/ERK1/2 Pathway in Head and Neck Squamous Cell Carcinoma. Oncol Rep (2018) 40(5):2558–72. doi: 10.3892/or.2018.6657 PMC6151899 30132555

[47] Cai J Xia L Li J Ni S Song H Wu X. Tumor-Associated Macrophages Derived TGF-β-Induced Epithelial to Mesenchymal Transition in Colorectal Cancer Cells Through Smad2,3-4/Snail Signaling Pathway. Cancer Res Treat (2019) 51(1):252–66. doi: 10.4143/crt.2017.613 PMC6333993 29690747

[48] Zhu L Fu X Chen X Han X Dong P. M2 Macrophages Induce EMT Through the TGF-β/Smad2 Signaling Pathway. Cell Biol Int (2017) 41(9):960–8. doi: 10.1002/cbin.10788 28493530

[49] Fan QM Jing YY Yu GF Kou XR Ye F Gao L. Tumor-Associated Macrophages Promote Cancer Stem Cell-Like Properties *via* Transforming Growth Factor-Beta1-Induced Epithelial-Mesenchymal Transition in Hepatocellular Carcinoma. Cancer Lett (2014) 352(2):160–8. doi: 10.1016/j.canlet.2014.05.008 24892648

[50] Wei C Yang C Wang S Shi D Zhang C Lin X. Crosstalk Between Cancer Cells and Tumor Associated Macrophages is Required for Mesenchymal Circulating Tumor Cell-Mediated Colorectal Cancer Metastasis. Mol Cancer (2019) 18(1):64. doi: 10.1186/s12943-019-0976-4 30927925 PMC6441214

[51] Cohen EN Gao H Anfossi S Mego M Reddy NG Debeb B. Inflammation Mediated Metastasis: Immune Induced Epithelial-To-Mesenchymal Transition in Inflammatory Breast Cancer Cells. PloS One (2015) 10(7):e0132710. doi: 10.1371/journal.pone.0132710 26207636 PMC4514595

[52] Chen Q Yang D Zong H Zhu L Wang L Wang X. Growth-Induced Stress Enhances Epithelial-Mesenchymal Transition Induced by IL-6 in Clear Cell Renal Cell Carcinoma *via* the Akt/GSK-3β/β-Catenin Signaling Pathway. Oncogenesis (2017) 6(8):e375. doi: 10.1038/oncsis.2017.74 28846080 PMC5608922

[53] Faget J Groeneveld S Boivin G Sankar M Zangger N Garcia M. Neutrophils and Snail Orchestrate the Establishment of a Pro-Tumor Microenvironment in Lung Cancer. Cell Rep (2017) 21(11):3190–204. doi: 10.1016/j.celrep.2017.11.052 29241546

[54] Huergo-Zapico L Parodi M Cantoni C Lavarello C Fernández-Martínez JL Petretto A. NK-Cell Editing Mediates Epithelial-To-Mesenchymal Transition *via* Phenotypic and Proteomic Changes in Melanoma Cell Lines. Cancer Res (2018) 78(14):3913–25. doi: 10.1158/0008-5472.CAN-17-1891 29752261

[55] Visciano C Liotti F Prevete N Cali' G Franco R Collina F. Mast Cells Induce Epithelial-to-Mesenchymal Transition and Stem Cell Features in Human Thyroid Cancer Cells Through an IL-8-Akt-Slug Pathway. Oncogene (2015) 34(40):5175–86. doi: 10.1038/onc.2014.441 25619830

[56] Min H Sun X Yang X Zhu H Liu J Wang Y. Exosomes Derived From Irradiated Esophageal Carcinoma-Infiltrating T Cells Promote Metastasis by Inducing the Epithelial-Mesenchymal Transition in Esophageal Cancer Cells. Pathol Oncol Res: POR (2018) 24(1):11–8. doi: 10.1007/s12253-016-0185-z 28132116

[57] Yu Y Xiao CH Tan LD Wang QS Li XQ Feng YM. Cancer-Associated Fibroblasts Induce Epithelial-Mesenchymal Transition of Breast Cancer Cells Through Paracrine TGF-β Signalling. Br J Cancer (2014) 110(3):724–32. doi: 10.1038/bjc.2013.768 PMC3915130 24335925

[58] Pistore C Giannoni E Colangelo T Rizzo F Magnani E Muccillo L. DNA Methylation Variations are Required for Epithelial-to-Mesenchymal Transition Induced by Cancer-Associated Fibroblasts in Prostate Cancer Cells. Oncogene (2017) 36(40):5551–66. doi: 10.1038/onc.2017.159 28581528

[59] Shintani Y Fujiwara A Kimura T Kawamura T Funaki S Minami M. IL-6 Secreted From Cancer-Associated Fibroblasts Mediates Chemoresistance in NSCLC by Increasing Epithelial-Mesenchymal Transition Signaling. J Thorac Oncol: Off Publ Int Assoc Study Lung Cancer (2016) 11(9):1482–92. doi: 10.1016/j.jtho.2016.05.025 27287412

[60] Zhao L Ji G Le X Luo Z Wang C Feng M. An Integrated Analysis Identifies STAT4 as a Key Regulator of Ovarian Cancer Metastasis. Oncogene (2017) 36(24):3384–96. doi: 10.1038/onc.2016.487 28114283

[61] Balamurugan K. HIF-1 at the Crossroads of Hypoxia, Inflammation, and Cancer. Int J Cancer (2016) 138(5):1058–66. doi: 10.1002/ijc.29519 PMC4573780 25784597

[62] Liu H Chen C Zeng J Zhao Z Hu Q. MicroRNA-210-3p is Transcriptionally Upregulated by Hypoxia Induction and Thus Promoting EMT and Chemoresistance in Glioma Cells. PloS One (2021) 16(7):e0253522. doi: 10.1371/journal.pone.0253522 34197482 PMC8248614

[63] Joseph JP Harishankar MK Pillai A Devi A. Hypoxia Induced EMT: A Review on the Mechanism of Tumor Progression and Metastasis in OSCC. Oral Oncol (2018) 80:23–32. doi: 10.1016/j.oraloncology.2018.03.004 29706185

[64] Hao Y Baker D Ten Dijke P. TGF-β-Mediated Epithelial-Mesenchymal Transition and Cancer Metastasis. Int J Mol Sci (2019) 20(11):2767. doi: 10.3390/ijms20112767 PMC6600375 31195692

[65] Gomez-Puerto MC Iyengar PV García de Vinuesa A Ten Dijke P Sanchez-Duffhues G. Bone Morphogenetic Protein Receptor Signal Transduction in Human Disease. J Pathol (2019) 247(1):9–20. doi: 10.1002/path.5170 30246251 PMC6587955

[66] Chang L Jia S Fu Y Zhou T Cao J He Q. Ougan (Citrus Reticulata Cv. Suavissima) Flavedo Extract Suppresses Cancer Motility by Interfering With Epithelial-to-Mesenchymal Transition in SKOV3 Cells. Chin Med (2015) 10:14. doi: 10.1186/s13020-015-0042-0 26131016 PMC4486131

[67] Bhowmick NA. Tgf-β Signaling in Fibroblastic Cells and Oncogenesis. Humana Press (2008) I:185–98. doi: 10.1007/978-1-59745-292-2_12

[68] Yang Y Pan X Lei W Wang J Song J. Transforming Growth Factor-Beta1 Induces Epithelial-to-Mesenchymal Transition and Apoptosis *via* a Cell Cycle-Dependent Mechanism. Oncogene (2006) 25(55):7235–44. doi: 10.1038/sj.onc.1209712 16799646

[69] Yang Y Pan X Lei W Wang J Shi J Li F. Regulation of Transforming Growth Factor-Beta 1-Induced Apoptosis and Epithelial-to-Mesenchymal Transition by Protein Kinase A and Signal Transducers and Activators of Transcription 3. Cancer Res (2006) 66(17):8617–24. doi: 10.1158/0008-5472.CAN-06-1308 16951175

[70] Bastos LG de Marcondes PG de-Freitas-Junior JC Leve F Mencalha AL de Souza WF. Progeny From Irradiated Colorectal Cancer Cells Acquire an EMT-Like Phenotype and Activate Wnt/β-Catenin Pathway. J Cell Biochem (2014) 115(12):2175–87. doi: 10.1002/jcb.24896 25103643

[71] Dong Z Zhou L Han N Zhang M Lyu X. Wnt/β-Catenin Pathway Involvement in Ionizing Radiation-Induced Invasion of U87 Glioblastoma Cells. Strahlentherapie und Onkologie (2015) 191(8):672–80. doi: 10.1007/s00066-015-0858-7 26072169

[72] Aoki M Fujishita T. Oncogenic Roles of the PI3K/AKT/mTOR Axis. Curr Topics Microbiol Immunol (2017) 407:153–89. doi: 10.1007/82_2017_6 28550454

[73] Chang L Graham PH Hao J Bucci J Cozzi PJ Kearsley JH. Emerging Roles of Radioresistance in Prostate Cancer Metastasis and Radiation Therapy. Cancer Metastasis Rev (2014) 33(2-3):469–96. doi: 10.1007/s10555-014-9493-5 24445654

[74] Yuan Y Liao H Pu Q Ke X Hu X Ma Y. miR-410 Induces Both Epithelial-Mesenchymal Transition and Radioresistance Through Activation of the PI3K/mTOR Pathway in non-Small Cell Lung Cancer. Signal Transduct Target Ther (2020) 5(1):85. doi: 10.1038/s41392-020-0182-2 32528035 PMC7290026

[75] Mao L Zhao ZL Yu GT Wu L Deng WW Li YC. γ-Secretase Inhibitor Reduces Immunosuppressive Cells and Enhances Tumour Immunity in Head and Neck Squamous Cell Carcinoma. Int J Cancer (2018) 142(5):999–1009. doi: 10.1002/ijc.31115 29047105

[76] Natsuizaka M Whelan KA Kagawa S Tanaka K Giroux V Chandramouleeswaran PM. Interplay Between Notch1 and Notch3 Promotes EMT and Tumor Initiation in Squamous Cell Carcinoma. Nat Commun (2017) 8(1):1758. doi: 10.1038/s41467-017-01500-9 29170450 PMC5700926

[77] Nowell CS Radtke F. Notch as a Tumour Suppressor. Nat Rev Cancer (2017) 17(3):145–59. doi: 10.1038/nrc.2016.145 28154375

[78] Wu SL Li YJ Liao K Shi L Zhang N Liu S. 2-Methoxyestradiol Inhibits the Proliferation and Migration and Reduces the Radioresistance of Nasopharyngeal Carcinoma CNE-2 Stem Cells *via* NF-κb/HIF-1 Signaling Pathway Inactivation and EMT Reversal. Oncol Rep (2017) 37(2):793–802. doi: 10.3892/or.2016.5319 28000883

[79] Ferrandino F Grazioli P Bellavia D Campese AF Screpanti I Felli MP. Notch and NF-κb: Coach and Players of Regulatory T-Cell Response in Cancer. Front Immunol (2018) 9:2165. doi: 10.3389/fimmu.2018.02165 30364244 PMC6193072

[80] Gao X Sun J Huang C Hu X Jiang N Lu C. RNAi-Mediated Silencing of NOX4 Inhibited the Invasion of Gastric Cancer Cells Through JAK2/STAT3 Signaling. Am J Trans Res (2017) 9(10):4440–9. PMC5666053 29118906

[81] Liu WH Chen MT Wang ML Lee YY Chiou GY Chien CS. Cisplatin-Selected Resistance is Associated With Increased Motility and Stem-Like Properties *via* Activation of STAT3/Snail Axis in Atypical Teratoid/Rhabdoid Tumor Cells. Oncotarget (2015) 6(3):1750–68. doi: 10.18632/oncotarget.2737 PMC4359329 25638155

[82] Wang J Hu W Du X Sun Y Han S Tu G. Fingolimod Inhibits Proliferation and Epithelial-Mesenchymal Transition in Sacral Chordoma by Inactivating IL-6/STAT3 Signalling. Biosci Rep (2020) 8(14):3824–40. doi: 10.1042/BSR20200221 PMC7029154 32027356

[83] Lu Z Wang J Zheng T Liang Y Yin D Song R. FTY720 Inhibits Proliferation and Epithelial-Mesenchymal Transition in Cholangiocarcinoma by Inactivating STAT3 Signaling. BMC Cancer (2014) 14:783. doi: 10.1186/1471-2407-14-783 25344679 PMC4221672

[84] Wang JX Yang Y Li K. Long Noncoding RNA DANCR Aggravates Retinoblastoma Through miR-34c and miR-613 by Targeting MMP-9. J Cell Physiol (2018) 233(10):6986–95. doi: 10.1002/jcp.26621 29744877

[85] Park YR Kim SL Lee MR Seo SY Lee JH Kim SH. MicroRNA-30a-5p (miR-30a) Regulates Cell Motility and EMT by Directly Targeting Oncogenic TM4SF1 in Colorectal Cancer. J Cancer Res Clin Oncol (2017) 143(10):1915–27. doi: 10.1007/s00432-017-2440-4 PMC11819409 28528497

[86] Bucay N Bhagirath D Sekhon K Yang T Fukuhara S Majid S. A Novel microRNA Regulator of Prostate Cancer Epithelial-Mesenchymal Transition. Cell Death Differentiation (2017) 24(7):1263–74. doi: 10.1038/cdd.2017.69 PMC5520174 28498363

[87] Baumann M Krause M Hill R. Exploring the Role of Cancer Stem Cells in Radioresistance. Nat Rev Cancer (2008) 8(7):545–54. doi: 10.1038/nrc2419 18511937

[88] Mondal S Bhattacharya K Mandal C. Nutritional Stress Reprograms Dedifferention in Glioblastoma Multiforme Driven by PTEN/Wnt/Hedgehog Axis: A Stochastic Model of Cancer Stem Cells. Cell Death Discovery (2018) 4:110. doi: 10.1038/s41420-018-0126-6 30534418 PMC6281623

[89] Fan CW Chen T Shang YN Gu YZ Zhang SL Lu R. Cancer-Initiating Cells Derived From Human Rectal Adenocarcinoma Tissues Carry Mesenchymal Phenotypes and Resist Drug Therapies. Cell Death Dis (2013) 4(10):e828. doi: 10.1038/cddis.2013.337 24091671 PMC3824647

[90] Yan C Luo L Goto S Urata Y Guo CY Doi H. Enhanced Autophagy in Colorectal Cancer Stem Cells Does Not Contribute to Radio-Resistance. Oncotarget (2016) 7(29):45112–21. doi: 10.18632/oncotarget.8972 PMC5216709 27129175

[91] Karandish F Froberg J Borowicz P Wilkinson JC Choi Y Mallik S. Peptide-Targeted, Stimuli-Responsive Polymersomes for Delivering a Cancer Stemness Inhibitor to Cancer Stem Cell Microtumors. Colloids Surfaces B Biointerfaces (2018) 163:225–35. doi: 10.1016/j.colsurfb.2017.12.036 PMC5818288 29304437

[92] Srivastava AK Banerjee A Cui T Han C Cai S Liu L. Inhibition of miR-328-3p Impairs Cancer Stem Cell Function and Prevents Metastasis in Ovarian Cancer. Cancer Res (2019) 79(9):2314–26. doi: 10.1158/0008-5472.CAN-18-3668 PMC6777340 30894370

[93] Atkins RJ Stylli SS Kurganovs N Mangiola S Nowell CJ Ware TM. Cell Quiescence Correlates With Enhanced Glioblastoma Cell Invasion and Cytotoxic Resistance. Exp Cell Res (2019) 374(2):353–64. doi: 10.1016/j.yexcr.2018.12.010 30562483

[94] Das PK Islam F Lam AK. The Roles of Cancer Stem Cells and Therapy Resistance in Colorectal Carcinoma. Cells (2020) 9(6):1392. doi: 10.3390/cells9061392 PMC7348976 32503256

[95] Wang P Wan WW Xiong SL Feng H Wu N. Cancer Stem-Like Cells can be Induced Through Dedifferentiation Under Hypoxic Conditions in Glioma, Hepatoma and Lung Cancer. Cell Death Discovery (2017) 3:16105. doi: 10.1038/cddiscovery.2016.105 28179999 PMC5253691

[96] Sancho P Burgos-Ramos E Tavera A Bou Kheir T Jagust P Schoenhals M. MYC/PGC-1α Balance Determines the Metabolic Phenotype and Plasticity of Pancreatic Cancer Stem Cells. Cell Metab (2015) 22(4):590–605. doi: 10.1016/j.cmet.2015.08.015 26365176

[97] Wang D Plukker J Coppes RP. Cancer Stem Cells With Increased Metastatic Potential as a Therapeutic Target for Esophageal Cancer. Semin Cancer Biol (2017) 44:60–6. doi: 10.1016/j.semcancer.2017.03.010 28366541

[98] Yokoi E Mabuchi S Shimura K Komura N Kozasa K Kuroda H. Lurbinectedin (PM01183), a Selective Inhibitor of Active Transcription, Effectively Eliminates Both Cancer Cells and Cancer Stem Cells in Preclinical Models of Uterine Cervical Cancer. Invest New Drugs (2019) 37(5):818–27. doi: 10.1007/s10637-018-0686-6 30374654

[99] Ishiwata T. Cancer Stem Cells and Epithelial-Mesenchymal Transition: Novel Therapeutic Targets for Cancer. Pathol Int (2016) 66(11):601–8. doi: 10.1111/pin.12447 27510923

[100] Morel AP Lièvre M Thomas C Hinkal G Ansieau S Puisieux A. Generation of Breast Cancer Stem Cells Through Epithelial-Mesenchymal Transition. PloS One (2008) 3(8):e2888. doi: 10.1371/journal.pone.0002888 18682804 PMC2492808

[101] Rasheed ZA Yang J Wang Q Kowalski J Freed I Murter C. Prognostic Significance of Tumorigenic Cells With Mesenchymal Features in Pancreatic Adenocarcinoma. J Natl Cancer Institute (2010) 102(5):340–51. doi: 10.1093/jnci/djp535 PMC2831049 20164446

[102] Kong D Banerjee S Ahmad A Li Y Wang Z Sethi S. Epithelial to Mesenchymal Transition is Mechanistically Linked With Stem Cell Signatures in Prostate Cancer Cells. PloS One (2010) 5(8):e12445. doi: 10.1371/journal.pone.0012445 20805998 PMC2929211

[103] Fan F Samuel S Evans KW Lu J Xia L Zhou Y. Overexpression of Snail Induces Epithelial-Mesenchymal Transition and a Cancer Stem Cell-Like Phenotype in Human Colorectal Cancer Cells. Cancer Med (2012) 1(1):5–16. doi: 10.1002/cam4.4 23342249 PMC3544430

[104] Long H Xiang T Qi W Huang J Chen J He L. CD133+ Ovarian Cancer Stem-Like Cells Promote non-Stem Cancer Cell Metastasis *via* CCL5 Induced Epithelial-Mesenchymal Transition. Oncotarget (2015) 6(8):5846–59. doi: 10.18632/oncotarget.3462 PMC4467406 25788271

[105] Olivares-Urbano MA Griñán-Lisón C Marchal JA Núñez MI. CSC Radioresistance: A Therapeutic Challenge to Improve Radiotherapy Effectiveness in Cancer. Cells (2020) 9(7):1651. doi: 10.3390/cells9071651 PMC7407195 32660072

[106] Gomez-Casal R Bhattacharya C Ganesh N Bailey L Basse P Gibson M. Non-Small Cell Lung Cancer Cells Survived Ionizing Radiation Treatment Display Cancer Stem Cell and Epithelial-Mesenchymal Transition Phenotypes. Mol Cancer (2013) 12(1):94. doi: 10.1186/1476-4598-12-94 23947765 PMC3751356

[107] Shi Y Liu N Lai W Yan B Chen L Liu S. Nuclear EGFR-PKM2 Axis Induces Cancer Stem Cell-Like Characteristics in Irradiation-Resistant Cells. Cancer Lett (2018) 422:81–93. doi: 10.1016/j.canlet.2018.02.028 29477380

[108] Tahmasebi-Birgani MJ Teimoori A Ghadiri A Mansoury-Asl H Danyaei A Khanbabaei H. Fractionated Radiotherapy Might Induce Epithelial-Mesenchymal Transition and Radioresistance in a Cellular Context Manner. J Cell Biochem (2018) 120(5):8601–10. doi: 10.1002/jcb.28148 30485518

[109] Gupta PB Pastushenko I Skibinski A Blanpain C Kuperwasser C. Phenotypic Plasticity: Driver of Cancer Initiation, Progression, and Therapy Resistance. Cell Stem Cell (2019) 24(1):65–78. doi: 10.1002/jcb.28148 30554963 PMC7297507

[110] Roma-Rodrigues C Mendes R Baptista PV Fernandes AR. Targeting Tumor Microenvironment for Cancer Therapy. Int J Mol Sci (2019) 20(4):840. doi: 10.3390/ijms20040840 PMC6413095 30781344

[111] Kachikwu EL Iwamoto KS Liao YP DeMarco JJ Agazaryan N Economou JS. Radiation Enhances Regulatory T Cell Representation. Int J Radiat Oncol Biol Phys (2011) 81(4):1128–35. doi: 10.1016/j.ijrobp.2010.09.034 PMC3117954 21093169

[112] Qu Y Jin S Zhang A Zhang B Shi X Wang J. Gamma-Ray Resistance of Regulatory CD4+CD25+Foxp3+ T Cells in Mice. Radiat Res (2010) 173(2):148–57. doi: 10.1667/RR0978.1 20095846

[113] Barker HE Paget JT Khan AA Harrington KJ. The Tumour Microenvironment After Radiotherapy: Mechanisms of Resistance and Recurrence. Nat Rev Cancer (2015) 15(7):409–25. doi: 10.1038/nrc3958 PMC4896389 26105538

[114] Tsoutsou PG Zaman K Martin Lluesma S Cagnon L Kandalaft L Vozenin MC. Emerging Opportunities of Radiotherapy Combined With Immunotherapy in the Era of Breast Cancer Heterogeneity. Front Oncol (2018) 8:609. doi: 10.3389/fonc.2018.00609 30619749 PMC6305124

[115] Takeshima T Pop LM Laine A Iyengar P Vitetta ES Hannan R. Key Role for Neutrophils in Radiation-Induced Antitumor Immune Responses: Potentiation With G-CSF. Proc Natl Acad Sci USA (2016) 113(40):11300–5. doi: 10.1073/pnas.1613187113 PMC5056034 27651484

[116] Park B Yee C Lee KM. The Effect of Radiation on the Immune Response to Cancers. Int J Mol Sci (2014) 15(1):927–43. doi: 10.3390/ijms15010927 PMC3907847 24434638

[117] Murakami S Yoshino H Ishikawa J Yamaguchi M Tsujiguchi T Nishiyama A. Effects of Ionizing Radiation on Differentiation of Murine Bone Marrow Cells Into Mast Cells. J Radiat Res (2015) 56(6):865–71. doi: 10.1093/jrr/rrv061 PMC4628224 26453633

[118] Müller K Meineke V. Radiation-Induced Mast Cell Mediators Differentially Modulate Chemokine Release From Dermal Fibroblasts. J Dermatol Sci (2011) 61(3):199–205. doi: 10.1016/j.jdermsci.2011.01.003 21292447

[119] Heissig B Rafii S Akiyama H Ohki Y Sato Y Rafael T. Low-Dose Irradiation Promotes Tissue Revascularization Through VEGF Release From Mast Cells and MMP-9-Mediated Progenitor Cell Mobilization. J Exp Med (2005) 202(6):739–50. doi: 10.1084/jem.20050959 PMC2212942 16157686

[120] Miyazaki T Ikeda K Sato W Horie-Inoue K Inoue S. Extracellular Vesicle-Mediated EBAG9 Transfer From Cancer Cells to Tumor Microenvironment Promotes Immune Escape and Tumor Progression. Oncogenesis (2018) 7(1):7. doi: 10.1038/s41389-017-0022-6 29362448 PMC5833691

[121] Fernström E Minta K Andreasson U Sandelius Å Wasling P Brinkmalm A. Cerebrospinal Fluid Markers of Extracellular Matrix Remodelling, Synaptic Plasticity and Neuroinflammation Before and After Cranial Radiotherapy. J Internal Med (2018) 284:211–25. doi: 10.1111/joim.12763 29664192

[122] Sangaletti S Tripodo C Santangelo A Castioni N Portararo P Gulino A. Mesenchymal Transition of High-Grade Breast Carcinomas Depends on Extracellular Matrix Control of Myeloid Suppressor Cell Activity. Cell Rep (2016) 17(1):233–48. doi: 10.1016/j.celrep.2016.08.075 27681434

[123] Tommelein J De Vlieghere E Verset L Melsens E Leenders J Descamps B. Radiotherapy-Activated Cancer-Associated Fibroblasts Promote Tumor Progression Through Paracrine IGF1R Activation. Cancer Res (2018) 78(3):659–70. doi: 10.1158/0008-5472.CAN-17-0524 29217764

[124] Nurmik M Ullmann P Rodriguez F Haan S Letellier E. In Search of Definitions: Cancer-Associated Fibroblasts and Their Markers. Int J Cancer (2020) 146(4):895–905. doi: 10.1002/ijc.32193 30734283 PMC6972582

[125] Bao CH Wang XT Ma W Wang NN Un Nesa E Wang JB. Irradiated Fibroblasts Promote Epithelial-Mesenchymal Transition and HDGF Expression of Esophageal Squamous Cell Carcinoma. Biochem Biophys Res Commun (2015) 458(2):441–7. doi: 10.1016/j.bbrc.2015.02.001 25677618

[126] Lin S Chen S Chen Z Dai Q Ke C. X-Ray-Induced Epithelial-Mesenchymal Transition in SW480 Colorectal Cancer Cells and its Potential Mechanisms. J B U ON: Off J Balkan Union Oncol (2017) 22(6):1457–62. 29332338

[127] Tsukamoto H Shibata K Kajiyama H Terauchi M Nawa A Kikkawa F. Irradiation-Induced Epithelial-Mesenchymal Transition (EMT) Related to Invasive Potential in Endometrial Carcinoma Cells. Gynecol Oncol (2007) 107(3):500–4. doi: 10.1016/j.ygyno.2007.08.058 17905419

[128] Lee SY Jeong EK Ju MK Jeon HM Kim MY Kim CH. Induction of Metastasis, Cancer Stem Cell Phenotype, and Oncogenic Metabolism in Cancer Cells by Ionizing Radiation. Mol Cancer (2017) 16(1):10. doi: 10.1186/s12943-016-0577-4 28137309 PMC5282724

[129] Gu H Huang T Shen Y Liu Y Zhou F Jin Y. Reactive Oxygen Species-Mediated Tumor Microenvironment Transformation: The Mechanism of Radioresistant Gastric Cancer. Oxid Med Cell Longevity (2018) 2018:5801209. doi: 10.1155/2018/5801209 PMC5892229 29770167

[130] Nagarajan D Melo T Deng Z Almeida C Zhao W. ERK/Gsk3β/Snail Signaling Mediates Radiation-Induced Alveolar Epithelial-to-Mesenchymal Transition. Free Radical Biol Med (2012) 52(6):983–92. doi: 10.1016/j.freeradbiomed.2011.11.024 PMC3288246 22198183

[131] Mahabir R Tanino M Elmansuri A Wang L Kimura T Itoh T. Sustained Elevation of Snail Promotes Glial-Mesenchymal Transition After Irradiation in Malignant Glioma. Neuro-oncology (2014) 16(5):671–85. doi: 10.1093/neuonc/not239 PMC3984547 24357458

[132] Cheng H Lee SH Wu S. Effects of N-Acetyl-L-Cysteine on Adhesive Strength Between Breast Cancer Cell and Extracellular Matrix Proteins After Ionizing Radiation. Life Sci (2013) 93(21):798–803. doi: 10.1016/j.lfs.2013.09.029 24113073 PMC3851573

[133] Pan Y Zhou C Yuan D Zhang J Shao C. Radiation Exposure Promotes Hepatocarcinoma Cell Invasion Through Epithelial Mesenchymal Transition Mediated by H2S/CSE Pathway. Radiat Res (2016) 185(1):96–105. doi: 10.1667/RR14177.1 26727544

[134] Rankin EB Giaccia AJ. Hypoxic Control of Metastasis. Sci (New York NY) (2016) 352(6282):175–80. doi: 10.1126/science.aaf4405 PMC4898055 27124451

[135] de Marcondes PG Morgado-Díaz JA. The Role of EphA4 Signaling in Radiation-Induced EMT-Like Phenotype in Colorectal Cancer Cells. J Cell Biochem (2017) 118(3):442–5. doi: 10.1002/jcb.25738 27632701

[136] Kuonen F Secondini C Rüegg C. Molecular Pathways: Emerging Pathways Mediating Growth, Invasion, and Metastasis of Tumors Progressing in an Irradiated Microenvironment. Clin Cancer Res: an Off J Am Assoc Cancer Res (2012) 18(19):5196–202. doi: 10.1158/1078-0432.CCR-11-1758 22730447

[137] Wang J Xu Z Wang Z Du G Lun L. TGF-Beta Signaling in Cancer Radiotherapy. Cytokine (2021) 148:155709. doi: 10.1016/j.cyto.2021.155709 34597918

[138] Andarawewa KL Erickson AC Chou WS Costes SV Gascard P Mott JD. Ionizing Radiation Predisposes Nonmalignant Human Mammary Epithelial Cells to Undergo Transforming Growth Factor Beta Induced Epithelial to Mesenchymal Transition. Cancer Res (2007) 67(18):8662–70. doi: 10.1158/0008-5472 17875706

[139] Zhou YC Liu JY Li J Zhang J Xu YQ Zhang HW. Ionizing Radiation Promotes Migration and Invasion of Cancer Cells Through Transforming Growth Factor-Beta-Mediated Epithelial-Mesenchymal Transition. Int J Radiat Oncol Biol Phys (2011) 81(5):1530–7. doi: 10.1016/j.ijrobp.2011.06.1956 22115555

[140] Li T Zeng ZC Wang L Qiu SJ Zhou JW Zhi XT. Radiation Enhances Long-Term Metastasis Potential of Residual Hepatocellular Carcinoma in Nude Mice Through TMPRSS4-Induced Epithelial-Mesenchymal Transition. Cancer Gene Ther (2011) 18(9):617–26. doi: 10.1038/cgt.2011.29 21637307

[141] Jung JW Hwang SY Hwang JS Oh ES Park S Han IO. Ionising Radiation Induces Changes Associated With Epithelial-Mesenchymal Transdifferentiation and Increased Cell Motility of A549 Lung Epithelial Cells. Eur J Cancer (Oxford England: 1990) (2007) 43(7):1214–24. doi: 10.1016/j.ejca.2007.01.034 17379505

[142] Ahmed KM Li JJ. NF-Kappa B-Mediated Adaptive Resistance to Ionizing Radiation. Free Radical Biol Med (2008) 44(1):1–13. doi: 10.1016/j.freeradbiomed.2007.09.022 17967430 PMC2266095

[143] Duru N Fan M Candas D Menaa C Liu HC Nantajit D. HER2-Associated Radioresistance of Breast Cancer Stem Cells Isolated From HER2-Negative Breast Cancer Cells. Clin Cancer Res an Off J Am Assoc Cancer Res (2012) 18(24):6634–47. doi: 10.1158/1078-0432.CCR-12-1436 PMC3593096 23091114

[144] Cao N Li S Wang Z Ahmed KM Degnan ME Fan M. NF-kappaB-Mediated HER2 Overexpression in Radiation-Adaptive Resistance. Radiat Res (2009) 171(1):9–21. doi: 10.1667/RR1472.1 19138055 PMC2659759

[145] Kim RK Kaushik N Suh Y Yoo KC Cui YH Kim MJ. Radiation Driven Epithelial-Mesenchymal Transition is Mediated by Notch Signaling in Breast Cancer. Oncotarget (2016) 7(33):53430–42. doi: 10.18632/oncotarget.10802 PMC5288197 27462787

[146] Huang X Borgström B Kempengren S Persson L Hegardt C Strand D. Breast Cancer Stem Cell Selectivity of Synthetic Nanomolar-Active Salinomycin Analogs. BMC Cancer (2016) 16:145. doi: 10.1186/s12885-016-2142-3 26906175 PMC4765157

[147] Kusunoki S Kato K Tabu K Inagaki T Okabe H Kaneda H. The Inhibitory Effect of Salinomycin on the Proliferation, Migration and Invasion of Human Endometrial Cancer Stem-Like Cells. Gynecol Oncol (2013) 129(3):598–605. doi: 10.1016/j.ygyno.2013.03.005 23500085

[148] Smigiel JM Parameswaran N Jackson MW. Potent EMT and CSC Phenotypes Are Induced By Oncostatin-M in Pancreatic Cancer. Mol Cancer Res: MCR (2017) 15(4):478–88. doi: 10.1158/1541-7786.MCR-16-0337 PMC5380554 28053127

[149] Jin Y Xu K Chen Q Wang B Pan J Huang S. Simvastatin Inhibits the Development of Radioresistant Esophageal Cancer Cells by Increasing the Radiosensitivity and Reversing EMT Process *via* the PTEN-PI3K/AKT Pathway. Exp Cell Res (2018) 362(2):362–9. doi: 10.1016/j.yexcr.2017.11.037 29208461

[150] Leiker AJ DeGraff W Choudhuri R Sowers AL Thetford A Cook JA. Radiation Enhancement of Head and Neck Squamous Cell Carcinoma by the Dual PI3K/mTOR Inhibitor PF-05212384. Clin Cancer Res an Off J Am Assoc Cancer Res (2015) 21(12):2792–801. doi: 10.1158/1078-0432.CCR-14-3279 PMC4470749 25724523

[151] Fury MG Lee NY Sherman E Ho AL Rao S Heguy A. A Phase 1 Study of Everolimus + Weekly Cisplatin + Intensity Modulated Radiation Therapy in Head-and-Neck Cancer. Int J Radiat Oncol Biol Phys (2013) 87(3):479–86. doi: 10.1016/j.ijrobp.2013.06.2043 24074921

[152] Narayan V Vapiwala N Mick R Subramanian P Christodouleas JP Bekelman JE. Phase 1 Trial of Everolimus and Radiation Therapy for Salvage Treatment of Biochemical Recurrence in Prostate Cancer Patients Following Prostatectomy. Int J Radiat Oncol Biol Phys (2017) 97(2):355–61. doi: 10.1016/j.ijrobp.2016.10.013 27986349

[153] Deutsch E Le Péchoux C Faivre L Rivera S Tao Y Pignon JP. Phase I Trial of Everolimus in Combination With Thoracic Radiotherapy in non-Small-Cell Lung Cancer. Ann Oncol Off J Eur Soc Med Oncol (2015) 26(6):1223–9. doi: 10.1093/annonc/mdv105 25701455

[154] Liu H Yang W Gao H Jiang T Gu B Dong Q. Nimotuzumab Abrogates Acquired Radioresistance of KYSE-150R Esophageal Cancer Cells by Inhibiting EGFR Signaling and Cellular DNA Repair. OncoTargets Ther (2015) 8:509–18. doi: 10.2147/OTT.S76958 PMC4348136 25750543

[155] Maddalo M Triggiani L Magrini SM Prof Buglione M. Cetuximab and Radiation Therapy in Head and Neck Cancer. Int J Radiat Oncol Biol Phys (2019) 105(3):678–9. doi: 10.1016/j.ijrobp.2019.07.025 31540600

[156] Takebe N Miele L Harris PJ Jeong W Bando H Kahn M. Targeting Notch, Hedgehog, and Wnt Pathways in Cancer Stem Cells: Clinical Update. Nat Rev Clin Oncol (2015) 12(8):445–64. doi: 10.1038/nrclinonc.2015.61 PMC4520755 25850553

[157] Zhang X Zheng L Sun Y Wang T Wang B. Tangeretin Enhances Radiosensitivity and Inhibits the Radiation-Induced Epithelial-Mesenchymal Transition of Gastric Cancer Cells. Oncol Rep (2015) 34(1):302–10. doi: 10.3892/or.2015.3982 25998143

[158] Diaz R Nguewa PA Redrado M Manrique I Calvo A. Sunitinib Reduces Tumor Hypoxia and Angiogenesis, and Radiosensitizes Prostate Cancer Stem-Like Cells. Prostate (2015) 75(11):1137–49. doi: 10.1002/pros.22980 25893276

[159] Lai KG Lin YH Ho CT Chen CY Peng CY Liu TZ. Paclitaxel Pretreatment Overcomes Hypoxia Inducible Factor-1α-Induced Radioresistance Acquisition of Human Hepatoma and Lung Adenocarcinoma Cells. Life Sci (2015) 136:7–12. doi: 10.1016/j.lfs.2015.06.006 26135626

[160] Lee JH Shim JW Choi YJ Heo K Yang K. The Combination of Sorafenib and Radiation Preferentially Inhibits Breast Cancer Stem Cells by Suppressing HIF-1α Expression. Oncol Rep (2013) 29(3):917–24. doi: 10.3892/or.2013.2228 PMC3597559 23314174

[161] Wang D Qin Q Jiang QJ Wang DF. Bortezomib Sensitizes Esophageal Squamous Cancer Cells to Radiotherapy by Suppressing the Expression of HIF-1α and Apoptosis Proteins. J X-Ray Sci Technol (2016) 24(4):639–46. doi: 10.3233/XST-160571 27080362

[162] Prasad P Gordijo CR Abbasi AZ Maeda A Ip A Rauth AM. Multifunctional Albumin-MnO₂ Nanoparticles Modulate Solid Tumor Microenvironment by Attenuating Hypoxia, Acidosis, Vascular Endothelial Growth Factor and Enhance Radiation Response. ACS Nano (2014) 8(4):3202–12. doi: 10.1021/nn405773r 24702320

[163] Lee K Zhang H Qian DZ Rey S Liu JO Semenza GL. Acriflavine Inhibits HIF-1 Dimerization, Tumor Growth, and Vascularization. Proc Natl Acad Sci USA (2009) 106(42):17910–5. doi: 10.1073/pnas.0909353106 PMC2764905 19805192

[164] Harada H Itasaka S Zhu Y Zeng L Xie X Morinibu A. Treatment Regimen Determines Whether an HIF-1 Inhibitor Enhances or Inhibits the Effect of Radiation Therapy. Br J Cancer (2009) 100(5):747–57. doi: 10.1038/sj.bjc.6604939 PMC2653764 19223896

[165] Gupta PB Onder TT Jiang G Tao K Kuperwasser C Weinberg RA. Identification of Selective Inhibitors of Cancer Stem Cells by High-Throughput Screening. Cell (2009) 138(4):645–59. doi: 10.1016/j.cell.2009.06.034 PMC4892125 19682730

[166] Thorne CA Hanson AJ Schneider J Tahinci E Orton D Cselenyi CS. Small-Molecule Inhibition of Wnt Signaling Through Activation of Casein Kinase 1α. Nat Chem Biol (2010) 6(11):829–36. doi: 10.1038/nchembio.453 PMC3681608 20890287

[167] Jang GB Hong IS Kim RJ Lee SY Park SJ Lee ES. Wnt/β-Catenin Small-Molecule Inhibitor CWP232228 Preferentially Inhibits the Growth of Breast Cancer Stem-Like Cells. Cancer Res (2015) 75(8):1691–702. doi: 10.1158/0008-5472.CAN-14-2041 25660951

[168] Gonsalves FC Klein K Carson BB Katz S Ekas LA Evans S. An RNAi-Based Chemical Genetic Screen Identifies Three Small-Molecule Inhibitors of the Wnt/wingless Signaling Pathway. Proc Natl Acad Sci USA (2011) 108(15):5954–63. doi: 10.1073/pnas.1017496108 PMC3076864 21393571

[169] Hirsch HA Iliopoulos D Tsichlis PN Struhl K. Metformin Selectively Targets Cancer Stem Cells, and Acts Together With Chemotherapy to Block Tumor Growth and Prolong Remission. Cancer Res (2009) 69(19):7507–11. doi: 10.1158/0008-5472.CAN-09-2994 PMC2756324 19752085

[170] Vazquez-Martin A Oliveras-Ferraros C Cufí S Del Barco S Martin-Castillo B Menendez JA. Metformin Regulates Breast Cancer Stem Cell Ontogeny by Transcriptional Regulation of the Epithelial-Mesenchymal Transition (EMT) Status. Cell Cycle (Georgetown Tex) (2010) 9(18):3807–14. doi: 10.4161/cc.9.18.13131 20890129

[171] Park JH Kim YH Park EH Lee SJ Kim H Kim A. Effects of Metformin and Phenformin on Apoptosis and Epithelial-Mesenchymal Transition in Chemoresistant Rectal Cancer. Cancer Sci (2019) 110(9):2834–45. doi: 10.1111/cas.14124 PMC6726705 31278880

